# scGWAS: landscape of trait-cell type associations by integrating single-cell transcriptomics-wide and genome-wide association studies

**DOI:** 10.1186/s13059-022-02785-w

**Published:** 2022-10-17

**Authors:** Peilin Jia, Ruifeng Hu, Fangfang Yan, Yulin Dai, Zhongming Zhao

**Affiliations:** 1 Center for Precision Health, School of Biomedical Informatics, The University of Texas Health Science Center at Houston, Houston, TX 77030 USA; 2 Human Genetics Center, School of Public Health, The University of Texas Health Science Center at Houston, Houston, TX 77030 USA; 3 MD Anderson Cancer Center UTHealth Graduate School of Biomedical Sciences, Houston, TX 77030 USA

**Keywords:** scGWAS, GWAS, Single-cell RNA sequencing, scRNA-seq, Complex diseases, Cell-type specificity

## Abstract

**Background:**

The rapid accumulation of single-cell RNA sequencing (scRNA-seq) data presents unique opportunities to decode the genetically mediated cell-type specificity in complex diseases. Here, we develop a new method, scGWAS, which effectively leverages scRNA-seq data to achieve two goals: (1) to infer the cell types in which the disease-associated genes manifest and (2) to construct cellular modules which imply disease-specific activation of different processes.

**Results:**

scGWAS only utilizes the average gene expression for each cell type followed by virtual search processes to construct the null distributions of module scores, making it scalable to large scRNA-seq datasets. We demonstrated scGWAS in 40 genome-wide association studies (GWAS) datasets (average sample size *N* ≈ 154,000) using 18 scRNA-seq datasets from nine major human/mouse tissues (totaling 1.08 million cells) and identified 2533 trait and cell-type associations, each with significant modules for further investigation. The module genes were validated using disease or clinically annotated references from ClinVar, OMIM, and pLI variants.

**Conclusions:**

We showed that the trait-cell type associations identified by scGWAS, while generally constrained to trait-tissue associations, could recapitulate many well-studied relationships and also reveal novel relationships, providing insights into the unsolved trait-tissue associations. Moreover, in each specific cell type, the associations with different traits were often mediated by different sets of risk genes, implying disease-specific activation of driving processes. In summary, scGWAS is a powerful tool for exploring the genetic basis of complex diseases at the cell type level using single-cell expression data.

**Supplementary Information:**

The online version contains supplementary material available at 10.1186/s13059-022-02785-w.

## Background

Nearly 90% of the disease susceptibility loci reported in genome-wide association studies (GWAS) are located in non-coding regions and are predicted to play regulatory roles [1]. However, genetic regulation is highly tissue- and cell-type-specific [2–6]. Identification of the genetically mediated associations between traits and cell types is critical to understand the functional impact of genetic variants and the underlying disease mechanisms, which can be further extended for potential precision medicine strategies. Such tasks have been complicated because multiple cell types are often associated with most complex traits. These cell types may or may not involve the same tissues. Furthermore, the cells in which the disease-susceptibility variants play regulatory roles may not be the cells that are most relevant to the disease symptoms [7]. Trait and cell type associations also have implications for disease complications and dynamic progression. Thus, an unbiased approach, rather than an experience-based or a priori knowledge-guided way, is urgently needed to quantify how the GWAS-implied genes are concordantly activated in a particular cell type.

Several studies have explored trait and cell-type associations, especially for inferring cell-type-specific gene expression patterns. For example, genes implicated in neurological diseases or psychiatric disorders were actively expressed in different types of neurons [8, 9]. By integrating mouse single-cell RNA sequencing (scRNA-seq) data with body mass index (BMI) GWAS, Timshel and colleagues reported that brain cell types were involved in obesity [10]. The method RolyPoly identifies the enrichment of SNP-trait association signals in functional annotations [11]. The online platform, Functional Mapping and Annotation of Genome-Wide Association Studies (FUMA), proposed a framework to map cell type specificity for complex traits [12, 13]. The method LD score regression applied to specifically expressed genes (LDSC-SEG) identified disease-relevant tissues and cell types by integrating gene expression data together with GWAS summary statistics [8, 14]. In our recent work, we conducted a systematic enrichment analysis for a wide variety of human traits to determine the trait-associated cell types in different organs [15, 16]. However, most of these works are enrichment-based analyses following a framework that, given a list of query genes, these methods determine if the query genes are significantly specifically expressed in a particular cell type. It remains unclear how disease-risk variants and related genes are transcriptionally activated in each cell type and further disrupt specific biological processes to affect disease risks.

Single-cell RNA sequencing technique can quantitatively measure gene expression at the resolution of individual cells and examine cell type-specific transcriptome features. Such information can be integrated into GWAS analysis to discover cell-type specificities for human complex traits [15, 17]. Here, we propose *scGWAS* (scRNA-seq assisted GWAS analysis) to investigate the transcriptional changes of genetic variants in specific cell type contexts by leveraging a wide variety of gene–gene relationships in the human genome. scGWAS can not only identify the genetically mediated associations between cell types and traits but also construct the biological networks that are overrepresented with disease risk genes and transcriptionally active genes in a cell type. As shown below, scGWAS utilizes only the average gene expression for each cell type, which makes it scalable to large scRNA-seq datasets. The reported associations between traits and cell types represent credible concordances between the two data types, regardless of the contrasts with other cell types. With such a design, we collected 18 scRNA-seq datasets from nine major human tissues (blood, brain, decidua, esophagus, heart, liver, lung, pancreas, and spleen) and applied scGWAS to 40 GWAS summary datasets of representative complex traits and disorders. We conducted comprehensive validation by using clinically annotated references such as ClinVar [18], OMIM [19], and pLI variants [20]. With the comprehensive map of trait and cell type associations, we further explored the driving processes contributing to the association pairs in several major disease groups such as metabolic diseases and immune-related diseases.

## Results

### The trait-tissue-cell type relationship

**Fig. 1:**
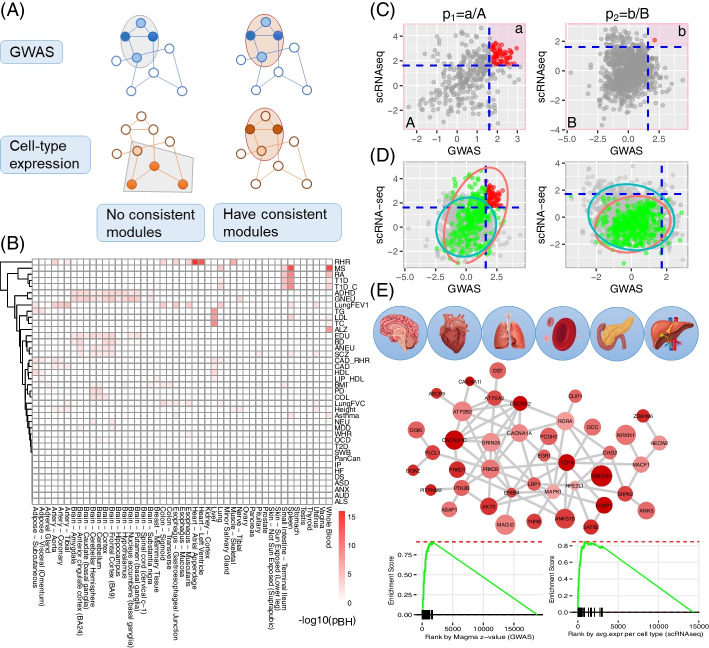
Analysis framework to decode trait-associated tissues and cell types. **A** Illustration of GWAS and cell-type expression integration at the cellular level. **B** Tissue-specific enrichment analysis of the traits. The color reflects the significance level [− log_10_(p_BH_)]. **C** Demonstration of the proportional test. **D** Demonstration of a case showing the association between GWAS and cell type transcriptome (left) and another case without such an association (right). In each figure, a dot indicates a module, with its GWAS-based score shown on the *x*-axis and its scRNA-seq score shown on the *y*-axis. The gray dots are random modules from the randomization process. The green and red dots are modules from the real data whereas the red ones indicate significance. Cyan and red circles indicate the 95% confidence interval (CI) of the random modules and the actual modules, respectively. The horizontal and vertical dash lines indicate nominal significance (*z* = 1.96). E Illustration of disease subnetworks and the enrichment result of the component genes in each of the two heterogeneous data sets

We collected 40 GWAS summary statistics datasets for representative complex traits (average sample size *N* ≈ 154,000), including psychiatric disorders, neurodegenerative disorders, immune diseases, metabolism traits, and others (Table 1). We conducted an initial investigation of the tissue specificity of these traits using our method *deTS* and the GTEx bulk transcriptome data [21]. As shown in Fig. 1B, a number of trait-tissue associations were identified, most of which were consistent with biological expectations, such as neuropsychiatric and cognitive traits enriched in different brain regions, immune-related traits enriched in spleen and whole blood, and lipid metabolic traits enriched in liver. A few traits [anxiety (ANX), internalizing problems (IP), obsessive–compulsive disorder (OCD), and pancreatic cancer (PanCan)] showed no association with any tissue. Notably, four tissues (whole blood, lung, spleen, and small intestine terminal ileum) were consistently associated with several diseases common in immune regulation, such as type 1 diabetes (T1D), multiple sclerosis (MS), and rheumatoid arthritis (RA), though some associations lacked obvious biological links, e.g., RA with small intestine terminal ileum. Therefore, to better understand the active context of these traits, cell-type level analysis is critically needed to fine-map the associations at the cellular level.

**Table 1:** Summary of complex traits/diseases and implicated tissues

Trait/disease name	Abbr	Year	# samples	Implied tissue	*p*_BH_(deTS)
Alcohol use disorder [22]	AUD	2019	202,004	Brain—cerebellum	0.08
Alzheimer’s disease [23]	AD	2018	455,258	Whole blood	9.94 × 10^−9^
Amyotrophic lateral sclerosis [24]	ALS	2016	36,052	Heart—atrial appendage	0.28
Anxiety, anxiety-continuous [25]	ANX	2016	18,186	Brain—spinal cord (cervical c-1)	0.10
Anxiety tension-special-factor-of-neuroticism[26]	ANEU	2019	270,059	Brain—anterior cingulate cortex (BA24)	6.49 × 10^−3^
Asthma [27]	Asthma	2017	127,669	Spleen	9.71 × 10^−3^
Attention-deficit-hyperactivity disorder [28]	ADHD	2017	53,293	Brain—anterior cingulate cortex (BA24)	9.05 × 10^−4^
PGC Autism, Autism-Europeans [29]	ASD	2017	13,574	Heart—atrial appendage	0.40
Bipolar disorder [30]	BD	2018	51,710	Brain—anterior cingulate cortex (BA24)	6.85 × 10^−4^
Blood lipids, high-density lipoprotein [31]	HDL	2010	99,900	Liver	6.62 × 10^−4^
Blood lipids, low-density lipoprotein [31]	LDL	2010	95,454	Liver	2.11 × 10^−8^
Blood lipids, total cholesterol [31]	TC	2010	100,184	Liver	6.91 × 10^−10^
Blood lipids, triglycerides [31]	TG	2010	96,598	Liver	1.31 × 10^−8^
Body mass index [32]	BMI	2015	234,069	Colon—sigmoid	0.02
CAD resting heart rate [33]	CAD_RHR	2016	265,046	Liver	4.80 × 10^−3^
Coronary artery disease [34]	CAD	2017	63,731	Artery—aorta	0.01
Depressive symptoms [35]	DS	2019	181,045	Adrenal gland	0.18
Educational attainment, education years all [36]	EDU	2016	293,723	Brain—frontal cortex (BA9)	3.31 × 10^−4^
General factor of neuroticism [26]	GNEU	2019	270,059	Brain—nucleus accumbens (basal ganglia)	3.10 × 10^−5^
Heart failure [37]	HF	2018	394,156	Artery—aorta	0.19
Height [38]	Height	2014	253,288	Artery—tibial	0.02
Internalizing problems [39]	IP	2014	4596	Adrenal gland	0.44
Lipoprotein concentrations, HDL [40]	LIP_HDL	2009	19,840	Adipose—visceral (omentum)	0.03
Lung function, FEV1/FVC [41]	FEV1	2019	316,614	Artery—tibial	1.52 × 10^−5^
Lung function, FVC [41]	FVC	2019	317,222	Colon—sigmoid	1.04 × 10^−3^
Major depressive disorder [42]	MDD	2018	42,455	Brain—anterior cingulate cortex (BA24)	0.04
Multiple sclerosis [43]	MS	2018	41,505	Spleen	9.03 × 10^−15^
Neuroticism [35]	NEU	2019	523,783	Brain—cerebellar hemisphere	9.18 × 10^−3^
Obsessive–compulsive disorder [44]	OCD	2017	9,725	Brain—cerebellum	0.05
Pancreatic cancer [45]	PanCan	2009	3,576	Adipose—subcutaneous	0.73
Parkinson’s disease [46]	PD	2012	8,477	Brain—cerebellum	3.65 × 10^−4^
Resting heart rate [47]	RHR	2019	458,969	Heart—atrial appendage	9.27 × 10^−17^
Rheumatoid arthritis [48]	RA	2014	58,284	Spleen	2.80 × 10^−10^
Schizophrenia [49]	SCZ	2018	74,626	Brain—frontal cortex (BA9)	6.60 × 10^−3^
SSGAC College [50]	COL	2013	101,069	Brain—cerebellar hemisphere	2.76 × 10^−3^
Subjective wellbeing [51]	SWB	2016	298,420	Brain—amygdala	0.20
Type 2 diabetes [52]	T2D	2017	159,208	Brain—spinal cord (cervical c-1)	0.10
Type 1 diabetes [53]	T1D	2011	26,890	Spleen	8.64 × 10^−9^
Type 1 diabetes, childhood adiposity age under17 [54]	T1D_C	2017	14,741	Spleen	7.61 × 10^−10^
Waist format 2: Waist hip ratio [55]	WHR	2015	143,480	Esophagus—Muscularis	0.04

*PGC* Psychiatric Genomics Consortium

### Methodology design of scGWAS

**Fig. 2:**
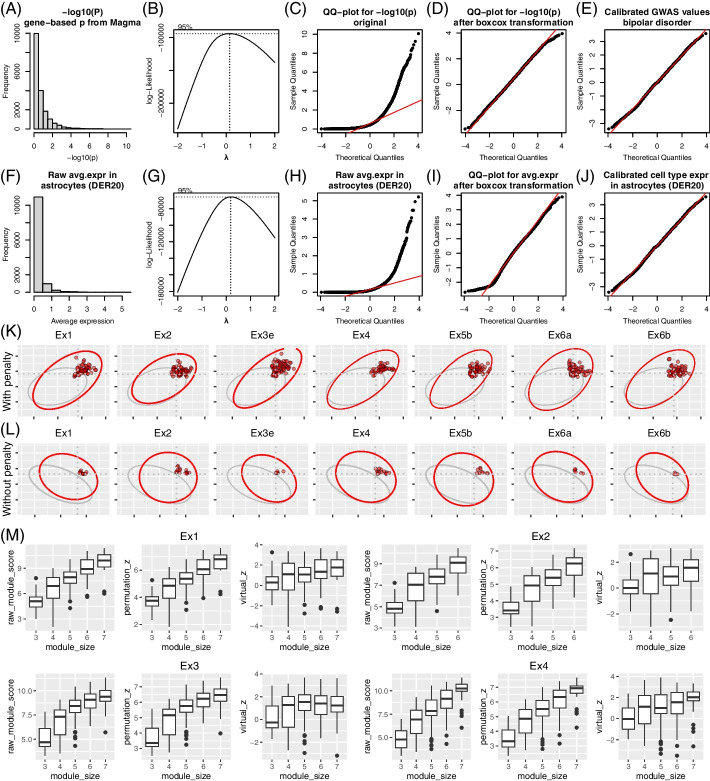
Illustration of the scGWAS method. **A**–**J** Illustration of the normalization process. **A** The distribution of gene-based *p*-values calculated from GWAS summary statistics, using bipolar disorder as an example. **B** Estimation of lambda values in Box-Cox transformation. **C** QQ plot of the original gene-based *p*-values from GWAS. **D** QQ plot of the Box-Cox transformed gene-based p-values. **E** QQ plot of the Box-Cox transformed and calibrated gene-based p-values. **F**–**J** Distribution and QQ plot of the gene expression for the astrocyte cell type in the DER20 panel, in the same order as **A**–**E**. **K**, **L** Illustration using seven cell types from the DER22 panel to show the impact of the penalty factor, where the top panel shows modules identified including the penalty factor (**K**) and the bottom panel shows modules identified excluding the penalty factor (**L**). A full comparison using all cell types can be found in Additional file 1: Fig. S2. **M** Comparison of different normalization methods for module scores. For each cell type, we show three types of module score distribution: the raw module score, permutation-based *z*-score, and the *z*-score based on size-matched random modules from the virtual search process. More comparison examples can be found in Additional file 1: Fig. S3

The principle of scGWAS is illustrated in Fig. 1A and more details are in the “Methods” section. Briefly, scGWAS has two goals: to determine if GWAS-implied genes are concordantly activated in a particular cell type (through the proportional test) and to identify gene modules in which both genetic association signals and cell-type expression signals are significantly enriched (through module identification). In the design of scGWAS, there are several important steps to ensure the accuracy of the results. First, we propose a novel procedure to normalize the GWAS data and the scRNA-seq data such that they could be integrated. As illustrated in Fig. 2, both the original GWAS scores and cell type expression scores tended to be right-skewed. With the Box-Cox transformation, the original distribution of − log10 (p) from MAGMA and the distribution of log (CPM+1) from scRNA-seq could be transformed to follow the normal distribution (Fig. 2D, I). With the normalization step, both types of scores were calibrated to approximate the normal distribution (Fig. 2E, J). Second, we develop a sequential feedforward module expansion coupled with backward examination (MEBE) algorithm to construct gene modules overrepresented with the heterogeneous information weighted by GWAS and by cellular expression data. The introduction of the inclusion step (controlled by *r*1) and the exclusion step (controlled by *r*2) allows scGWAS to always retain informative nodes in the modules. We examined the random modules from the virtual search process and determined the values for *r*1 and *r*2 for the following analyses (Additional file 1: Fig. S1; see more discussion in Additional file 1 and in our previous works [17, 56]). Third, when executing MEBE, we define a module score *m* with a penalty factor $sd({m}_{g},{m}_{s})$ to control deviation of the two types of weights. Indeed, without the penalty factor, i.e., using $m={m}_{g}+{m}_{s}$ to calculate the module score, fewer modules were identified compared to the cases when we included the penalty factor (Fig. 2K, L and Additional file 1: Fig. S2). Including the factor also increased the chance to discover more disease and cell type associations that were reported in previous works, e.g., excitatory neurons with schizophrenia [8, 12]. Fourth, we introduce a process called virtual search to construct the null distribution of module scores as the theoretical parameters have proven difficult to estimate [57–59]. This virtual search process breaks down the relationship between GWAS signals and cell type expression while implementing the same module search procedure (i.e., the MEBE algorithm) repeatedly until a sufficient number of random modules have been generated to form the null distribution. These random modules are subsequently used to assess the significance of modules (*p*_*m*_) and to conduct the proportional test (i.e., to generate the *z*-score). As shown in Fig. 2M and Additional file 1: Fig. S3, this procedure reduced the effect of module sizes and made modules with different sizes comparable.

### Comparison of scGWAS with similar methods

There have been several similar methods for enrichment analyses of trait-associated SNPs in specific cells [11, 13–16]. Notably, most of these methods report the cell types that are relevant to the traits but they do not particularly report the genes that mediate the enrichment. Here, we compared scGWAS with FUMA [13]. As shown in Additional file 1: Fig. S4, scGWAS was able to predict trait and cell-type associations that were comparable to FUMA but it further identified the subnetworks to infer how each component gene has mediated the association. More discussion on the technical details of the method comparison can be found in Additional file 1.

### scGWAS identified trait-cell type associations that recapitulated biological expectations

**Fig. 3:**
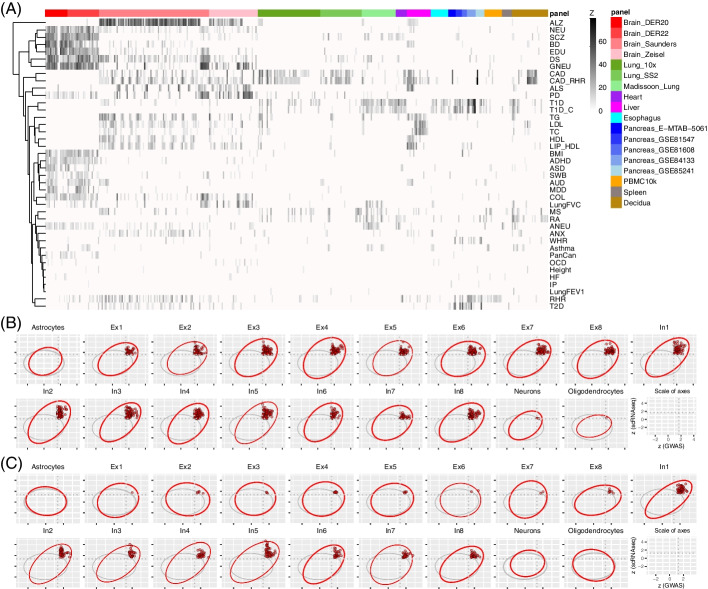
Illustration of the scGWAS results. **A** A heatmap of all scGWAS results using 18 scRNA-seq panels and 40 traits. **B**, **C** Demonstration of module score distribution in schizophrenia (**B**) and major depressive disorder (**C**) in cell types from the DER20 panel. The last plot shows the scale of the axes: normalized module score from scRNA-seq on the *y*-axis and normalized module score from GWAS on the *x*-axis. In each panel, the red circle indicates the 95% confidence interval (CI) estimated using the random modules and the blue circle indicates the 95% CI estimated using the real modules. Significant modules are highlighted in red while all other modules, including non-significant modules from real data and all random modules from the virtual runs, are not plotted for simplicity

We applied scGWAS to the 40 GWAS summary statistics using all 18 single-cell panels. These panels reported a total of 437 tissue-cell types, including shared cell types (such as immune, epithelial, and stroma cells) among tissues and unique cell types that were tissue-specific (such as acinar, delta, ductal, and gamma cells in pancreas and neurons primarily in the nervous system) (Additional file 1: Table S1). Throughout this work, we refer to a cell type pertaining to a specific panel rather than merging cells of the same type across different panels to avoid the potential introduction of batch effect. For example, the B cells were reported in the lung, blood, heart, or spleen panels and we specified this cell type when necessary. For each pair of a trait and a cell type, we tested whether the proportion of concordant modules was significantly higher than randomly expected through the proportional test. As a result, we conducted 40 × 437 times of runs and identified a total of 2533 trait-cell type associations for which a significant association was identified (proportional test, z > 5) (Fig. 3). Significant modules were also reported (*p*_*m*_ < 0.05). The cutoff of *z* > 5 was determined according to the Bonferroni correction for 437 cells and 40 traits (Φ^−1^(0.05/437 × 40) ≈ 5). Among the 18 panels, the four brain panels revealed the greatest number of associations (Saunders: *n* = 806; Zeisel: *n* = 290; DER22: *n* = 327; DER20: *n* = 236). This is likely due to a large number of cell types per panel and also the large proportion of brain disorders investigated (20 out of 40 traits). Accordingly, several cell types that had the largest number of associated traits were from the four brain panels: microglia, two excitatory neurons (Ex2 and Ex8), and astrocyte. Among all traits, resting heart rate in coronary artery disease (CAD_RHR) had the largest number of associations (*n* = 166 tissue-cell types), followed by depressive symptoms (DS, *n* = 133), Alzheimer’s disease (ALZ, *n* = 125), coronary artery disease (CAD, *n* = 115), type 1 diabetes (T1D, *n* = 109), and resting heart rate (RHR, *n* = 108). Four traits had a much smaller number of associations: height (*n* = 9), heart failure (HF, *n* = 8), lung function FEV1-FVC (*n* = 4), and internalizing problems (IP, *n* = 3), which were consistent with the observations at the tissue level. All the remaining traits had 10–100 trait and cell type associations.

Of note, the associations we found confirmed many previous discoveries. Among the 40 tested GWAS summary datasets, the traits with significant associations with brain cell types were almost exclusively brain-related traits and disorders and they formed two sub-clusters (Fig. 3), one with NEU, SCZ, BD, EDU, DS, and GNEU, and the other with BMI, ADHD, ASD, SWB, AUD, MDD, and COL (see Table 1 for abbreviations). Our results confirmed the association between BMI and several neuronal cell types, including both excitatory neurons and inhibitory neurons in both panels. This is consistent with previous studies that obesity was related to brain tissues and cell types [10]. Plasma lipid traits [i.e., high-density lipoprotein (HDL), low-density lipoprotein (LDL), total cholesterol (TC), and triglycerides (TG)] were found in liver cells. Diabetes [type 2 diabetes (T2D), type 1 diabetes (T1D), and type 1 diabetes with childhood adiposity (T1D_C)] were associated with the pancreatic tissue and consistently with the beta cell type in multiple panels. Finally, immune-related traits, including those with immune-related dysfunctions [e.g., rheumatoid arthritis (RA) and multiple sclerosis (MS)], were found with immune cells from various panels. Apart from these expected associations, novel associations were identified, which implied potential comorbidity mechanisms. For example, we found CAD and CAD_RHR with many cell types in the lungs, consistent with previous reports that lung impairment is associated with coronary artery disease [60] and RHR with beta cells from the pancreas [61].

### scGWAS reports more clinically relevant genes

**Fig. 4:**
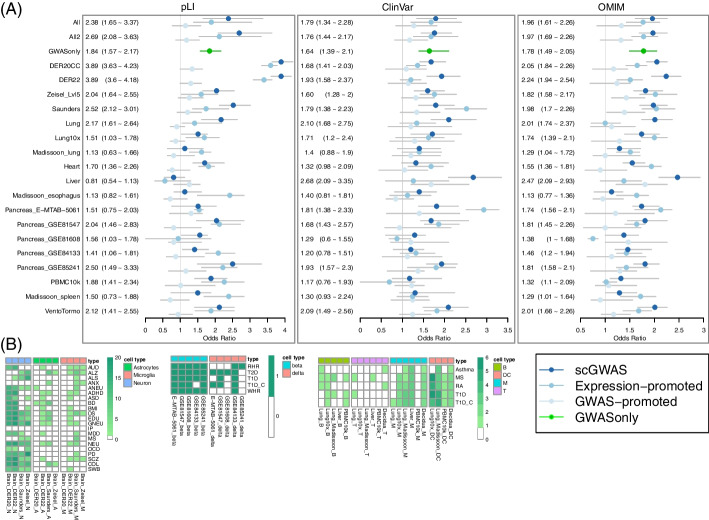
Independent validation of scGWAS results. **A** Validation of module genes using pLI, ClinVar, and OMIM annotations. For each scRNA-seq panel, we showed the forest plot using three sets (the scGWAS-identified module gene set, the GWAS-promoted set, and the expression-promoted set) using the mean OR with the 25–75% range. The values on the left part of each plot were the mean OR (25–75% OR) for module genes identified by scGWAS. All: module gene sets from all the trait and cell-type associations. All2: module gene sets with ≥ 20 genes. We also showed the results for module genes identified by using only GWAS data (GWASonly). **B** Cross-panel validation of the trait and cell-type associations. Brain_DER20_N, Brain_DER20_A, and Brain_DER20_M are short for the neuron, astrocytes, and microglia cell types in the DER20 panel. Brain_DER20_E and Brain_DER20_I refer to the excitatory and inhibitory neurons in the DER20 panel. Lung10x_B, Lung10x_T, Lung10x_M, and Lung10x_DC refer to the B cells (including subtypes), T cells, macrophages, and dendritic cells in the Lung10x panel

We performed a series of enrichment analyses to evaluate the module genes identified by scGWAS. To this end, we downloaded (1) genes with pLI annotations based on the ExAC dataset, (2) ClinVar dataset in which genes were annotated with pathogenic or likely pathogenic variants, and (3) OMIM genes. For each trait-cell type association, we defined two gene sets as control: one contained the most highly expressed genes in the corresponding cell type expression data and had the same number of genes as the module genes; the other contained those that were ranked as the most significant in the corresponding GWAS data with the same size. For each gene set, we calculated the proportion of clinically relevant genes, defined by pLI (those with pLI > 0.9), ClinVar, or OMIM, in both the investigated gene set (module genes or control genes) and the reference gene set (containing the remaining genes). As shown in Fig. 4, our module genes outperformed most of the GWAS-promoted genes and the expression-promoted genes in the majority of tissues and in all three functionally important gene sets: pLI [in 17/18 panels, the scGWAS sets had an average odds ratio (OR) greater than the GWAS-promoted sets and in 11/18 panels greater than the expression-promoted sets], ClinVar (in 16/18 panels scGWAS sets greater than the GWAS-promoted sets and in 13/18 panels greater than the expression-promoted sets), and OMIM (in 17/18 panels scGWAS sets greater than the GWAS-promoted sets and in 11/18 panels greater than the expression-promoted sets). In addition, we implemented the same module search and virtual search process by using only GWAS data to construct subnetworks for each trait (referred to as GWAS_only). As shown in Fig. 4A, we found that overall, the module genes identified by scGWAS tended to be more functionally important than the module genes identified by using GWAS only (the average OR for pLI: 2.38 by scGWAS compared to 1.84 by GWAS only; 1.79 versus 1.64 for ClinVar; and 1.96 versus 1.78 for OMIM). Collectively, scGWAS was demonstrated to enrich more functional genes than the raw GWAS-promoted genes simply selected using the smallest *p-*values. These results provided convincing evidence that the module genes are more likely to be functionally important.

### scGWAS uncovers replicable trait-cell associations

The multiple traits and panels allowed us to perform a cross-panel evaluation of the results. We selected several cell types that were reported by multiple panels for cross-panel replication of the trait-cell associations, mainly cells from the brain, pancreas, and the immune system. For the brain panels, we focused on three general groups, i.e., neuron and major non-neuron cells (astrocyte and microglia), each of which had individual cell types reported in different panels. For the 20 brain-related disorders plus BMI, 19/19 traits in neuron, 8/15 traits in astrocyte, and 8/17 traits in microglia had replicated associations (≥ 2 panels). For neurons, three panels also distinguished inhibitory neurons from excitatory neurons and for these panels, 16 out of 19 traits were found in excitatory neurons by two or more panels and 16/19 were repeatedly found in the inhibitory neurons. For the pancreas, we examined beta and delta cells that were found associated with several traits. As shown in Fig. 4B, 5/5 and 4/5 were repeatedly found in all five panels. In addition, we examined the general groups of B cells, T cells, macrophage, and dendritic cells (DC). Different subtypes of these cells were found in the lung, spleen, liver, and decidua. Four traits that were previously reported with an immune component were repeatedly found associated with some or all of these immune-related cell types. Taken together, these cross-panel comparisons indicated that the associations found by scGWAS were reliable and replicable.

**Fig. 5:**
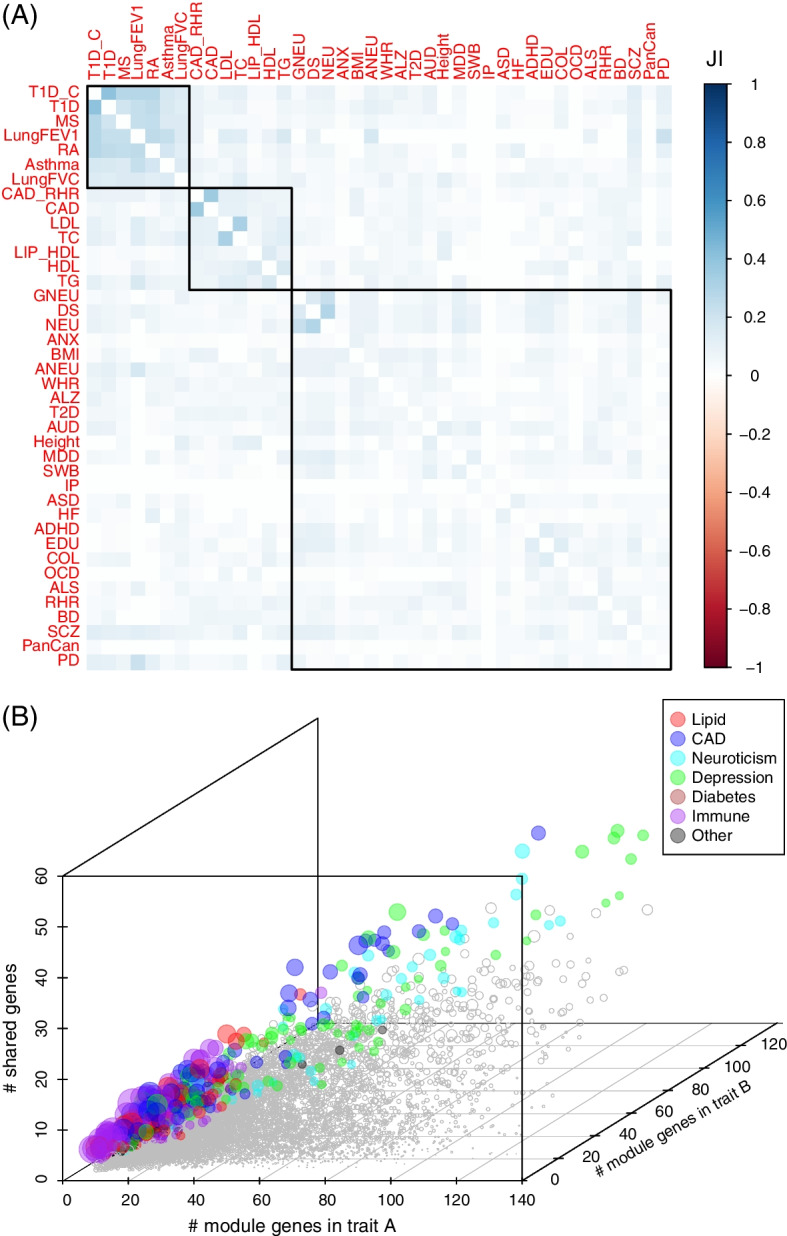
Investigation of the correlation among traits based on module genes. **A** Trait-trait correlation based on shared module genes across significantly enriched cell types. **B** Bubble plot of the trait-trait correlation in each cell type. Each dot represents a trait-trait correlation in a particular cell type. The size of the dot is proportional to the Jaccard Index (JI) based on module genes

It is worth noting the presence of batch effect among these datasets, as discussed before and in benchmarking analysis [62]. In our work, we did not conduct any preprocessing to control the batch effect except the built-in functions of scGWAS for normalization between GWAS and scRNA-seq. However, the results from our applications of scGWAS were consistent in detecting the associated cell types, implying the robustness of scGWAS to overcome batch effects and accurately detect trait-associated cell types.

### Cell-type-based trait similarity

Considering that multiple traits or disorders could be associated with the same cell types, we next investigated the shared genes among the investigated traits. We first collected the associated traits for each cell type and calculated the Jaccard Index (JI) based on the module genes. The similarity of any pair of traits was calculated as the mean JI across cell types. As shown in Fig. 5A, three major trait groups were identified: immune (asthma, T1D, T1D_C, MS, and RA), lipid (TG, HDL, LIP_HDL, and TC), and brain disorders (GNEU, DS, NEU, ALS, etc.). Through examining the pairs of traits that showed high overlapping genes (Fig. 5B), we found that the traits in the same group generally had more shared genes with each other than that with the traits from unrelated groups. Among the 575 pairs with a high JI value (> 0.2, accounting for 5.5% of all pairs), 94 between DS and NEU/GNEU, 89 occurred between CAD and CAD_RHR, 77 pairs belonged to the plasma lipid traits, 45 between NEU and GNEU, 202 belonged to the five immune-related traits (asthma, MS, RA, T1D, T1D_C), and the remaining among others. These results indicated that the traits sharing genetic components tended to have more module genes in the same cell type.

### Trait and cell type associations in the pancreas, liver, and the immune system

#### Diabetes and obesity-related traits in the pancreas

**Fig. 6:**
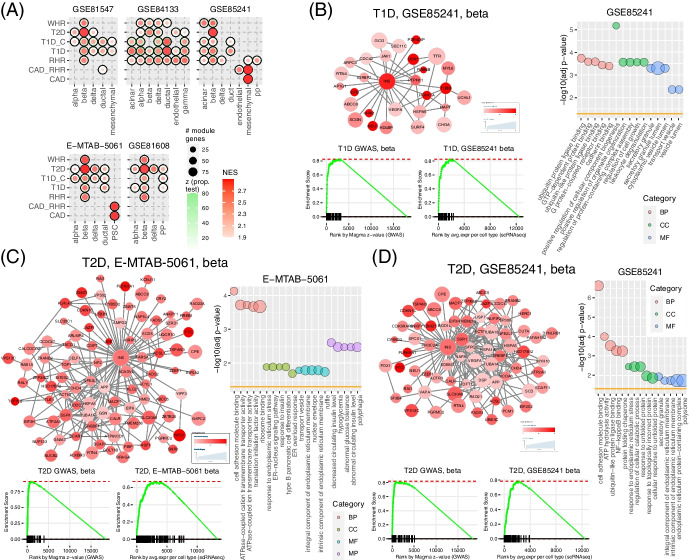
Trait-cell type association using the pancreas panels. **A** Association results using the five pancreas panels. **B**–**D** Demonstration and comparison of three subnetworks: T1D in the beta cell from the GSE85241 panel (**B**), T2D in the beta cell from the E-MTAB-5061 panel (**C**), and T2D in the beta cell from the GSE85241 panel (**D**). In all networks, node color is proportional to the corresponding GWAS signals and node size is proportional to the average gene expression in the corresponding cell type

The pancreas is the organ for maintaining metabolic balance in the human body. It is associated with several diseases, especially diabetes. scGWAS consistently reported that T2D was associated with the beta cells in all five scRNA-seq panels and also in the alpha and delta cells. Both T1D and T1D_C were found enriched in multiple cells including beta cells and delta cells (Fig. 6). Interestingly, resting heart rate and waist-hip ratio were also found enriched in beta cells in multiple datasets. The module genes that mediate the association between T1D and T2D in the beta cells had distinct functions, implying mechanistic insights underlying these diseases. T1D was mainly enriched in leukocyte-related functions, consistent with the immune hypothesis of this disease. In contrast, T2D was enriched in hormone secretion, highlighting the genes with previously reported evidence, such as *HSD17B12 *[63], *INS*, *SLC30A8 *[64], *ABCC8 *[65], and *FTO *[66]. In all panels, the insulin encoding gene *INS* served as the hub to the resultant subnetworks (Fig. 6). We demonstrated the results using the subnetwork in the normal pancreas (GSE85241, Fig. 6D) and the one in the disease samples (E-MTAB-5061, Fig. 6C). The module genes found in the disease samples were significantly enriched in response to insulin [Benjamini and Hochberg corrected *p*-value (p_BH_) = 1.90 × 10^−4^], type B pancreatic cell differentiation (p_BH_ = 2.12 × 10^−4^), and pancreas development (p_BH_ = 2.56 × 10^−4^) whereas the module genes in the normal samples tended to be enriched in various endoplasmic reticulum-related processes.

#### Different traits associated with the liver

**Fig. 7:**
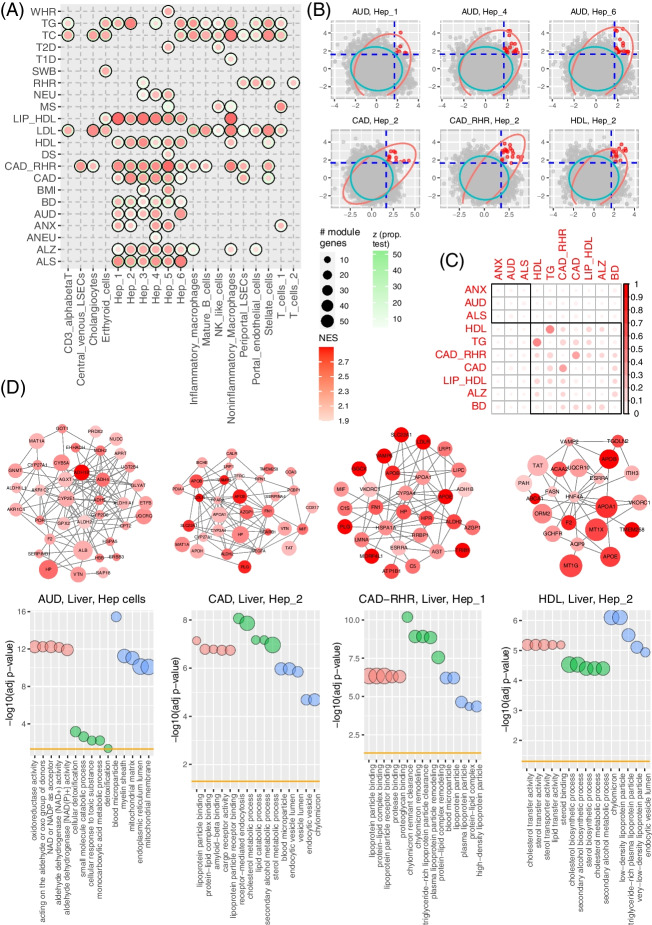
Trait-cell type association using the liver panel. **A** Heatmap of the identified trait-cell type associations in the liver panel. **B** Distribution of module scores using selected trait and cell type pairs as examples. In each panel, the cyan circle indicates the 95% confidence interval (CI) estimated using the random modules and the red circle indicates the 95% CI estimated using the real modules. Significant modules are highlighted in red while all other modules, including non-significant modules from real data and all random modules from the virtual runs, are plotted as gray dots. **C** Trait-trait correlation based on shared module genes in the Hep_1 cell. The red dots are proportional to the Jaccard Index between any pair of traits using their module genes identified in Hep_1. **D** Demonstration of subnetworks for AUD, CAD, CAD-RHR, and HDL. Note that the AUD network was constructed using all genes identified in Hep cells and the networks for CAD, CAD-RHR, and HDL were constructed using genes identified in a specific Hep cell type. In all networks, node color is proportional to the corresponding GWAS signals and node size is proportional to the average gene expression in the corresponding Hep cells

The liver is the major human tissue for metabolism, including ethanol metabolism [67]. Both AUD and lipid-related traits are well known to be associated with the liver. Using scGWAS, we found three groups of traits enriched in the liver hepatocyte (Hep) cells: brain related disorders (ALZ, ALS, AUD, BD, and NEU), lipid-related traits (LDL, HDL, TC, TG, and LIP_HDL), and cardiovascular traits (CAD and CAD_RHR) (Fig. 7). The AUD subnetwork included genes from the alcohol dehydrogenase family (e.g., *ADH1B*, *ADH4*, *ADH5*) and aldehyde dehydrogenase activity-related genes *ALDH1A1* and *ALDH2* (Fig. 7D), and those genes were enriched in many alcohol metabolic related processes such as alcohol dehydrogenase [NAD(P) +] activity (p_BH_ = 1.79 × 10^−5^) and alcohol catabolic process (p_BH_ = 6.40 × 10^−11^). In contrast, lipid-related traits were mainly enriched in lipoprotein-related functions. Interestingly, CAD and CAD_RHR were also enriched in the six hepatocyte cells in the liver, although the module genes in these traits highlighted cholesterol transfer activity (p_BH_ = 1.23 × 10^−46^), cholesterol metabolic process (p_BH_ = 1.65 × 10^−7^), and lipoprotein-related functions (Fig. 7). These results further demonstrated that different genes and processes are involved in different traits in the same cell type.

#### Immune-related traits with the lung

**Fig. 8:**
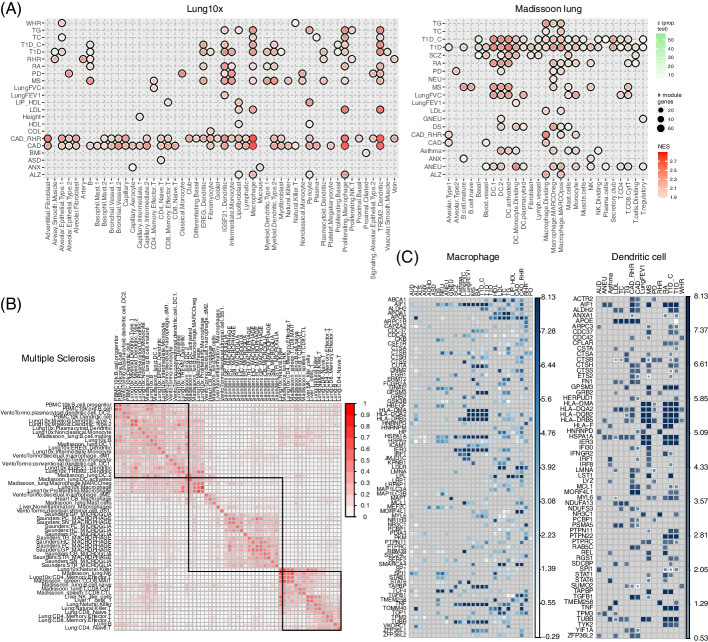
Trait-cell type association using the lung panels. **A** Heatmap of the identified trait-cell type associations in the Lung10x and Madissoon_Lung panels. **B** The cluster of cell types from different panels for MS. **C** Heatmap of genes that were frequently identified in different traits in the macrophage cells or the dendritic cells

The immune cells, and their various subtypes, existed in nearly all the panels we collected, especially the lung (Lung10x, LungSS2, Madissoon_Lung, Fig. 8, Additional file 1: Fig. S5), PBMC (Additional file 1: Fig. S5), spleen (Additional file 1: Fig. S5), and decidua (Additional file 1: Fig. S6). These included the major groups of B cells, T cells, macrophages, monocyte, and dendritic cells (DC). In our results, the traits/disorders with a strong immune component, such as asthma, MS, RA, T1D, and T1D_C, were frequently enriched in immune cells regardless of the tissue origin (Figs. 4B and 8A, and Additional file 1: Fig. S5). By examining the shared module genes, we found a number of HLA genes (e.g., *HLA-DMA*, *HLA-DQA2*, *HLA-DQB2*, *HLA-DRB5*, and *HLA-F*) as well as other genes (such as *TNF*) mediated the trait and immune cell associations (Fig. 8C). Interestingly, we also observed that heart diseases, i.e., CAD, CAD_RHR, and RHR, were frequently associated with many immune cell types from the lung, PBMC, and decidua. While these diseases were expected to be enriched in the heart, which were confirmed in our results (Additional file 1: Fig. S7), they were also associated with adventitial fibroblast, alveolar epithelial, and bronchial vessels in the lung. The cell types associated with these diseases in the decidua (Additional file 1: Fig. S6) and heart (Additional file 1: Fig. S7) were mainly stromal, endothelial, and fibroblast related. This observation supported the underlying disease etiology.

## Discussion

In this work, we proposed a novel method, scGWAS, to map genetic susceptibility genes from GWAS summary statistics to cell-type transcriptomes from healthy tissues. We applied scGWAS to 40 GWAS datasets covering a diverse set of complex human traits. Our results recapitulated previously known trait-tissue and trait-cell type associations and further revealed novel associations that have not been reported before. We observed that different sets of genes were activated in different cell types for the same diseases, implying complex disease-specific mechanisms. Overall, we presented a comprehensive landscape of trait and cell type associations as well as subnetworks for each association to further explore how the disease or trait susceptibility genes were specifically expressed in the corresponding cell type(s).

One of the advantages of scGWAS was that the trait and cell type associations were determined by using the average gene expression across all cells classified in a cell type. We did not conduct any cross-cell type comparison or define any measurement to assess the cell type specificity. Rather, we proposed a virtual search strategy to break down the pair-wise relationship between each node and its weights and construct the null distribution. As a result, the associations were not dependent on the comparison with other cell types from the scRNA-seq panel. Importantly, the association results represented the authentic concordance between cell type expression and the GWAS data. This design makes scGWAS widely applicable to various scRNA-seq data, either those with over one hundred cell types or as few as four (e.g., in the case of panel GSE81608). In addition, other forms of measurement can be chosen, e.g., gene expression variance (as highly variably expressed genes are often used to conduct classification analysis), cell-type specificity (as implemented in our previous work CSEA-DB [13] and deCS [16]), among others. Users can prepare different forms of cell type expression data as the input to scGWAS, with the hypothesis being updated to test the concordance between GWAS data with highly variably expressed genes (if the variance is used), or the concordance between GWAS data with highly specifically expressed genes (if the specificity scores are used).

There were several trait and cell type associations that we did not discuss in detail, but they were also supported by literature [15, 68]. These included T2D with the liver hepatocyte cells (Fig. 7A), ALS and ALZ with the liver hepatocyte cells (Fig. 7A), and several traits with the macrophage in the lung panels (Fig. 8A). Overall, the reported trait-cell type associations provided novel insights, facilitating further genetic studies, such as investigation of cell-type-specific regulation, annotation of epigenomics profiles for trait-associated candidate variants, and understanding the mechanisms of causal variants.

Throughout this work, we mainly used scRNA-seq data from normal tissues except for two disease panels from T2D patients (E-MTAB-5061 and GSE81608). This is because using scRNA-seq data from disease tissues would make it difficult to distinguish the impact of the original genetic background of the tissue itself and the impact of the investigated GWAS signals. It is also hard to determine whether the observed high gene activity in disease tissues is the reason or the result of the disease status. Using data from normal tissue, we could measure the gene expression activity without any disease confounders, and thus, they serve as an unbiased reference panel for GWAS data analyses.

There are some limitations of the current work. First, although scGWAS could identify a list of traits and cell type associations, it might have missed some true associations. For example, we failed to detect any associated cell type for pancreatic cancer, even though the appropriate tissue data for the pancreas were included. There were also panels in which the identified associations remained to be explained, such as the fetal heart panel, where we found ASD, AUD, asthma, and T1D were associated (Additional file 1: Fig. S6). Future work with expanded panels is necessary to complete the tissue annotations for cell types. Second, we ignored the co-expression relationships among gene–gene or protein–protein pairs when searching for modules. Incorporation of more specified gene–gene regulations, such as transcription factors and their target genes, may empower novel discoveries of regulatory module genes. Third, we used a generalized SNP to gene mapping strategy based on the physical location of each SNP. This strategy is limited to proximal regions and might miss important SNP-gene relationships. Comprehensive mapping of SNPs to genes using *cis*- and *trans*-annotations [e.g., eQTL, mQTL (methylation), histone-QTL, and pQTL (protein)] is expected to better explore the potential roles of SNPs with targeted genes. In practice, users can map SNPs to genes using customized strategies and then provide the resultant gene-based scores to scGWAS for follow-up analyses.

Lastly, there are several components in the method that are worthy of clarification. First, the module search process controls the concordance between GWAS weights and scRNA-seq weights through the penalty factor while the proportional test also assessed the concordance between the two data types. However, the proportional test is built on the true modules from the original data and the random modules from the virtual search process, with the latter being constructed through the same MEBE algorithm. As illustrated in the module score distribution (Figs. 2K and 3B, C), in random cases, even with the penalty factor, the two data types showed no association (i.e., the gray circle showing random modules is roughly in parallel with the *x*-axis and the *y*-axis). This indicates that the module search process does not introduce biases towards higher concordance itself and has no or very limited impact on the latter assessment by the proportional test. Thus, the GWAS data and scRNA-seq data were only used for module discovery but not for module evaluation; otherwise, it would raise an issue of overestimation of performance based on reusing the same data for two different tasks. Second, scGWAS makes no assumption about the reference network. Any type of gene–gene relationship can be used to serve as the reference network, e.g., co-expression, protein–protein interaction, genetic regulation, and so on. Accordingly, the interpretation of the scGWAS results will be adjusted by such reference networks. Third, scGWAS is not an end-to-end pipeline. For example, it does not include any module to preprocess, normalize, and batch correct single-cell RNA-seq data, nor to conduct cell type classification/clustering. Therefore, users need to use available single-cell pipelines, such as Seurat [69] and Scanpy [70], to define cell types and then to provide the annotations to scGWAS. Fourth, despite that our method takes single-cell level expression data as the input, scGWAS is not able to provide information on individual cells. Rather we leverage aggregation procedures (e.g., pseudo-bulk expression levels based on clusters or external annotations) as proposed by previously developed methods [11].

## Conclusions

In summary, to the best of our knowledge, the scGWAS method and the results in its application represent the first and most comprehensive investigation of trait GWAS data and cell type associations at the network modularity level, presenting a trait and cell type map for future studies.

## Methods

### GWAS data

The full names and references of the traits are available in Table 1. All GWAS data were obtained using samples of European ancestry. For each trait, we calculated gene-based *p*-value using Multi-marker Analysis of GenoMic Annotation (MAGMA, v1.07) [71]. SNPs that were located in the gene body or the flanking regions (50 kb upstream and 35 kb downstream) were included for each gene to calculate the gene-level *p*-values. Of note, mapping SNPs only in proximal regions to genes may miss important regulatory regions which can be located up to 1 Mb away from the gene. The 1000 Genomes Project Phase 3 European population was used as the reference panel to assess the linkage disequilibrium (LD) structure.

### scRNA-seq data

To identify the cell type specificity of diverse complex traits and phenotypes, we collected 18 scRNA-seq datasets (hereafter referred to as 18 panels) from 12 studies for 9 representative tissues that are typically involved in complex traits. They are peripheral blood mononuclear cells (panel name: PBMC10k) [72], human brain (DER20 [73–75], DER22 [73, 76]), mouse brain (Zeisel [77], Saunders [78]), decidua [79], esophagus [80], fetal heart [81], liver [82], lung (Lung10x [83], LungSS2 83], Madissoon_Lung [80]), pancreas (healthy pancreas: GSE81547 [84], GSE84133[85], GSE85241 [86]; healthy and T2D pancreas: E-MTAB-5061 [87] and GSE81608 [88]), and spleen [80]. In general, we excluded genes with low expression (i.e., those with expression value zero in more than 95% cells) and less represented cell types (i.e., expressed in < 30 cells), unless otherwise specified. We used the originally downloaded count data or UMI values to calculate counts per million (CPM), followed by log-transformation. For each cell type, the average log (CPM+1) value per gene was calculated to represent the cell-type transcriptome profile (Additional file 1: Table S1). The details of each dataset are presented in the Additional file 1.

## Construction of a working network with heterogeneous node weights

We collected the gene–gene relationship data from PathwayCommons [89] (v12, data access date: 12/12/2019) to construct the background network. The data downloaded originally had 1,851,006 interaction pairs that were curated and integrated from the public pathway and interaction databases. The relationships for these interactions included catalysis, chemical effect, regulation of expression or phosphorylation, react, and interacts-with, among others. We excluded those interactions that were annotated as *in-complex-with*, because those genes tended to be co-expressed and might inflate the results. We further excluded 2291 ribosomal genes and housekeeping genes defined by the HSIAO_HOUSEKEEPING_GENES set from MSigDB (expressed across 19 tissues [90]). In addition, for each gene–gene pair, we examined their genomic locations and excluded those pairs that were located within 50 kb of each other. Here 50 kb was the gene boundary region we used in the MAGMA analyses. This is to avoid duplicated information counting in the module search process when two interacting genes are physically close. Furthermore, we excluded all pairs whose interacting genes are located in the MHC region (chr6:26000000_34000000, hg19) due to the complex LD in this region. The resultant network served as the background network and was subsequently assigned with types of node weights: one from GWAS and the other from scRNA-seq. Each node had a weight defined by the GWAS signals (denoted by ${v}_{g}$), which was a normalized score based on − log10 transformed gene-based *p*-values, and a weight defined by the cell-type average expression (defined by ${v}_{s}$). Transformation and normalization were applied to make the two sets of weights compatible for integration (see below).

### Transformation of the raw data

The gene-based scores from GWAS theoretically should follow the standard normal distribution. Instead, the scores were found highly skewed and were likely driven by the local LD structure (e.g., the MHC region) or extremely significant loci (e.g., the APOE loci in Alzheimer’s disease). We thus apply the Box-Cox transformation using − log10 of gene-based *p*-values. The Box-Cox transformation provides a way to transform non-normal distribution to an approximately normal distribution. Specifically, given a query vector y =  − log10(*p*), where *p* is the gene-based *p*-value from MAGMA, the Box-Cox transformation is conducted as below: $$y\left(\lambda \right)=\left\{\begin{array}{c}\frac{{y}^{\lambda }-1}{\lambda }\text{, if }\lambda \ne 0\\ \mathrm{log}\left(y\right)\text{, if} \lambda =0\end{array}\right.$$. We search for the best lambda in the range of − 2 to 2 with a step of 0.05 that result in the best approximation of a normal distribution. When the best lambda is found 0, the second-best lambda will be selected. After this transformation, the gene-based scores from GWAS are approximately normal. We further shift the transformed values by the median value of the original data so that the new distribution has the same center. For scRNA-seq data, the log (CPM+1) values are transformed to approximate the normal distribution by using the same Box-Cox transformation strategy.

### Normalization of heterogeneous weights

To integrate the two heterogeneous sets of node weights, we next propose a normalization method to further calibrate the two distributions. Specifically, we first generate values using the rank-based inverse normal transformation (INT) for the same number of genes with weights. The INT values, which serve as the reference distribution, are then combined with the gene-based GWAS score and gene-based scRNA-seq score to form an *N* × 3 data matrix. We then apply the quantile normalization. In this way, both input scores are finely scaled and tend to approximate the normal distribution better. This process of calibration does not change the order of genes in either weight system, yet it aligns both weights to the normal distribution. Thus, it makes it fairly equivalent to combine the two weights. We also test by using scaling only, i.e., the regular *z*-score normalization. However, the extreme values present in either dataset would inflate the resultant modules, especially when the GWAS scores have strong *p*-values.

### Module score

We refer to the normalized node scores as ${{v}_{g}}^{(i)}$ and ${{v}_{s}}^{(i)}$ for the *i*^th^ node, respectively. The collection of ${{v}_{g}}^{(i)}$ (or ${{v}_{s}}^{(i)}, i=1,\dots ,N$, *N* is the total number of nodes) for all nodes in each GWAS and cell type pairs follow the standard normal distribution. We define the module score by integrating both the GWAS signals and the scRNA-seq expression: $m={m}_{g}+{m}_{s}-sd ({m}_{g},{m}_{s})$, where ${m}_{g}=\frac{{\sum }_{i}^{v}{{v}_{g}}^{(i)} }{\sqrt{|v|}}$ and ${m}_{s}=\frac{{\sum }_{i}^{v}{{v}_{s}}^{(i)} }{\sqrt{|v|}}$, and |*v|* is the number of nodes in the module. The part $sd \left({m}_{g},{m}_{s}\right)=\sqrt{{({m}_{g}-\frac{{m}_{g}+{m}_{s}}{2})}^{2}+{({m}_{s}-\frac{{m}_{g}+{m}_{s}}{2})}^{2}}$ functions as a penalty to control deviation of the GWAS signals and expression from each other such that the resultant modules are not overwhelmed by GWAS or expression individually. Thus, in the module search process, we aim to identify modules in which both GWAS and cell-type expression are highly scored.

## Module construction

We propose a sequential feedforward module expansion coupled with backward examination (MEBE) algorithm to identify modules. Briefly, we consider every node in the weighted network as a seed node and conduct module search, resulting in a module for each seed node. Starting with a seed gene, the module expands by recruiting the best neighbor node and also shrinks by trimming non-essential component genes. At each expansion step, the neighbor node that most improves the module score is added, if its addition also increases the module score by passing the predefined threshold, i.e., ${m}^{t+1}>{m}^{t}\times (1+r1)$, where $r1$ is the inclusion threshold. Upon expansion, a backward examination will be triggered to trim any leaf nodes that contribute minimally to the overall module score. That is, should the node be excluded, the decrease of the module score passes a predefined threshold, i.e., ${m}^{t-1}>{m}^{t}\times (1-r2)$, where $r2$ is the exclusion threshold. This expansion-trim combination continues until no more nodes in the neighborhood can improve the module score to the extent of $r1$ and no more nodes in the module make a marginal contribution as defined by $r2$. The trim step ensures that the final modules are concise, with those leaf nodes trimmed if their weights are marginal. Because of this expansion-trim design, different seed nodes may end up with the same module (i.e., with the same module genes) and the seed node may not necessarily be included in the final module.

### Virtual module search to construct the null distribution

In network analyses, the parameters of the null distribution have proven difficult to estimate [57–59]. To this end, we propose the virtual search strategy. In each round, we break down the relationship between nodes and their weights from GWAS as well as the relationship between nodes and their weights from scRNA-seq: $${v}^{(i)}\begin{array}{c}\nearrow \\ \searrow \end{array}\begin{array}{c}{{v}_{g}}^{(i)}\\ {{v}_{s}}^{(i)}\end{array}$$, where both relationships (denoted by the arrows) are broken down. However, we keep the structure of the reference network intact, where the edges remain the same. That is, the weights are permuted among the nodes in the graph, respectively for GWAS and for scRNA-seq data. We then run the MEBE algorithm on the randomly weighted network to generate modules that would subsequently be used to form the null distribution. In particular, module scores are normalized using the statistics from the null distribution formed by size-matched random modules to control the impact of module size. To this end, we keep repeating the virtual search process until all size-specific null distributions have sufficient numbers of random modules (i.e., $\ge$ 1000 for each module size). Both the real modules and random modules are first stratified based on the number of component genes. Module scores are normalized following the *z*-score transformation: ${z}_{m}=\frac{m-u}{sd}$, where $u$ and *sd* are the mean and standard deviation calculated using the random modules with the same module size. This process results in normalized module scores: ${z}_{m}$ for module score $m$, ${z}_{g}$ for ${m}_{g}$, and ${z}_{s}$ for ${m}_{s}$. Significant modules are defined as those with ${p}_{m}=\frac{\#(\pi >{z}_{m})}{\#\pi }<0.05$, ${p}_{g}=\frac{\#(\pi >{z}_{g})}{\#\pi }<0.05$, and ${p}_{s}=\frac{\#(\pi >{z}_{s})}{\#\pi }<0.05$, where $\pi$ is the collection of all random modules.

### Assessment of combined modules

We merge significant modules to form one final subnetwork for each investigated trait in each investigated cell type. Because different modules may share component genes, an overall assessment score is needed to measure the overall significance of the combined subnetwork. We employ the Gene Set Enrichment Analysis (GSEA) to calculate a normalized enrichment score (NES) using the GWAS data and the scRNA-seq data, respectively. The subnetwork NES is defined as the average value of the NES from GWAS and the NES from scRNA-seq.

### Assessment of concordance between GWAS and a cell-type transcriptome

We use the proportional test to assess whether the proportion of the concordant modules identified with the real data (${p}_{1}$) is significantly higher than that with the random data (${p}_{2}$). Here the concordant modules are defined by using both ${m}_{g}$ and ${m}_{s}$ to measure the concordance between GWAS and cell-type transcriptomes. We use the random modules from the virtual search to define two cutoff values to distinguish modules ranked within the top 5% of total modules according to either GWAS or scRNA-seq: $Q95\left({m}_{g}\right)$ for ${m}_{g}$ and $Q95\left({m}_{s}\right)$ for ${m}_{s}$ (the vertical and horizontal lines in red in Fig. 1). The former proportion is defined as ${p}_{1}=\frac{a}{A}=\frac{\# modules [{m}_{g}>Q95\left({m}_{g}\right) \& {m}_{s}>Q95\left({m}_{s}\right)]}{A}$ and the latter proportion is defined as ${p}_{2}=\frac{b}{B}=\frac{\# random modules [{m}_{g}>Q95\left({m}_{g}\right) \& {m}_{s}>Q95\left({m}_{s}\right)]}{B}$, where $A$ and $B$ are the total numbers of modules identified using the real data and random data, respectively. The pooled proportion is defined as $\widehat{p}=\frac{a+b}{A+B}$. A *z*-score is calculated as $z=\frac{{p}_{1}-{p}_{2}}{\sqrt{\widehat{p}\times (1-\widehat{p})\times (\frac{1}{A}+\frac{1}{B})}}$. A higher *z*-score indicates that the proportion in the real data is higher than that in the random data. More methodology details can be found in Additional file 1.

### Implementation

scGWAS is implemented in JAVA and is available to users as a JAR package. All other analyses were conducted using R. 

## Supplementary Information

Additional file 1: Additional information about data collection, data preprocess, and methodology design, aswell as additional Table S1 and Figures S1-S7.

Additional file 2: Review history.

## Data Availability

The scGWAS package is available at GitHub [91] and at the repository Zenodo [92] under a GNU General Public License v3.0. The GWAS summary statistics data can be downloaded following the references in Table 1. The scRNA-seq data can be downloaded following the references or URLs in Additional file 1: Table S1.
