# Supplementary material for: scGWAS: landscape of trait-cell type associations by integrating single-cell transcriptomics-wide and genome-wide association studies
Source: Genome Biol. 2022 Oct 17;23:220. doi: 10.1186/s13059-022-02785-w (PMC9575201; doi:10.1186/s13059-022-02785-w)
Supplement: Supplementary file 1 — Additional file 1: Additional information about data collection, data preprocess, and methodology design, aswell as additional Table S1 and Figures S1-S7. [file 13059_2022_2785_MOESM1_ESM.docx]

**Supplementary materials for “**scGWAS: landscape of trait-cell type associations by integrating single-cell transcriptomics-wide and genome-wide association studies”

**Single-cell RNA sequencing (scRNA-seq) data collection**

1. The Peripheral Blood Mononuclear Cells (PBMC) panel. The PBMC panel was collected from a healthy donor and was downloaded from the 10x genomics support website for single cell gene expression data^1^ (name: pbmc_10k_v3). In total, we collected 8682 genes for 14 cell types (CD4 memory, CD14 monocytes, NK dim, pre-B cell, NK bright, CD4 naïve, CD8 naïve, pDC, double negative T cell, CD16 monocytes, platelet, CD8 effector, B cell progenitor, and dendritic cell).
2. The human brain panel DER20. The DER20 dataset merged three studies: PsychENCODE (developmental)^2^, Darmanis et al. 2015^3^, and Lake et al. 2016^4^. There are 35 cell types reported in this panel from both fetal and adult brains, including 13 cell types from the fetal brain [astrocytes, endothelial, excitatory neuron (ExN), inhibitory neuron (IntN), intermediate progenitor cells (IPC), microglia, neuroepithelial cells (NEP), oligodendrocytes (Oligo), oligodendrocyte precursor cells (OPCs), pericytes, quiescent newly born neurons, replicating neuronal progenitors, and Transient cell type (trans)] and 22 cell types from the adult brain [astrocytes, endothelial, eight types of excitatory neurons (Ex1, Ex2, Ex3, Ex4, Ex5, Ex6, Ex7, and Ex8), eight subtypes of inhibitory neurons (In1, In2, In3, In4, In5, In6, In7, and In8), microglia, neurons, oligodendrocytes, and OPC]^2^. We excluded 7 cell types that had less than 30 cells (i.e., endothelial, fetal Oligo, fetal pericytes, fetal replicating, microglia, and OPC) as well as those lowly expressed genes, resulting in 14,306 genes in 28 cell types. Among them, 54.45% (in microglia) to 100% (in Ex1) genes were expressed in each cell type.
3. The human brain panel DER22. The DER22 dataset merged two studies from PsychENCODE (adult)^2^ and Lake et al. 2018^5^. It has 25 cell types, including 9 types of excitatory neurons (Ex1, Ex2, Ex3e, Ex4, Ex5b, Ex6a, Ex6b, Ex8, and Ex9), 10 inhibitory neurons (In1a, In1b, In1c, In3, In4a, In4b, In6a, In6b, In7, and In8), and astrocytes, endothelial, microglia, oligodendrocytes, OPC, and pericytes. A total of 27,380 cells were included in the dataset, with the cell type Per having the minimum number of cells (45) and Ex1 having the maximum number of cells (5059).
4. The mouse brain panel from Zeisel et al.^6^. The Zeisel study used scRNA-seq to systematically survey the mouse nervous system (19 regions) and reported a hierarchical organization of molecular cell types. The final, high-quality curated level of annotations included 265 clusters (i.e., cell types) grouped into 39 broad categories (level 4). In our work, we downloaded the expression matrix for the 265 cell types and further grouped them into the level 4 categories of 39 cell types. No cell type was filtered as they were defined in the original study. After removing genes with zero in >95% cells, we had 14848 genes in total, with 46.77% (cerebellum neurons) to 92.65% (Pericytes) expressed genes (non-zero expression) per cell type (median: 77.65%).
5. The mouse brain panel from Saunders et al.^7^. The Saunders study included single cells from 9 brain regions [frontal cortex (FC), striatum, globus pallidus externus/nucleus basalis (GP), thalamus (TH), hippocampus (HP), posterior cortex (PC), entopeduncular nucleus/subthalamic nucleus (EP), substantia nigra/ventral tegmental area (SN) and cerebellum (CB)] and reported 565 cell types. We grouped these cell types into 88 broad categories based on brain regions. Similar to the Zeisel panel, we kept all cell types as defined by the original study and excluded genes with zero in >95% cells, resulting in 21,277 genes in 88 cell types and each cell type expressing 35.40% (SN_MICROGLIA) to 99.90% (FC_NEURON) of the total genes.
6. The heart panel^8^. The human heart dataset included ~4000 cardiac cells from human embryos ranging from 5 weeks (5W) to 25 weeks of gestation^8^. We used the original category annotation for nine cell types: C1_5W, C2_CM (cardiomyocyte, CM), C3_Fibroblast-like cell (cardiac fibroblasts), C4_EC (endothelial cell, EC), C5_Valvar cell (valvar interstitial cell, VIC), C6_EP, C7_Mast cell, C8_Macrophage, and (C9_B/T cells). Only one cell type (C7_Mast cell) had 27 cells while all other eight cell types had >30 cells. Thus, we kept all nine cell types. After removing genes with zero in >95% cells, we had 13,095 genes for the following analyses.
7. The liver panel^9^. The liver dataset contained scRNA-seq profiles for 8444 parenchymal and non-parenchymal cells from five human livers^9^. We used the original category annotation for 20 discrete cell types, with Stellate cells having the smallest number of cells (37) and Hep_1 having the largest number of cells (1006). The liver panel contains organ specific cell types (e.g., the six hepatocytes: Hep_1, Hep_2, Hep_3, Hep_4, Hep_5, and Hep_6), immune cells (macrophages, B_cells, NK_like_cells, T_cells, etc.), and others. A total of 6717 genes were detected on this panel after excluding lowly expressed genes.
8. Three panels for lung (lungSS2 and lung10x). The lung datasets were profiled from ~75,000 human lung and blood cells prepared using two platforms: droplet-based 10x Chromium (10x) protocol or FACS-sorted cells followed by SmartSeq2 (SS2) protocol^10^. We processed these two datasets independently and referred to them as lungSS2 (33 cell types) and lung10x (54 cell types) respectively. Both lung panels contained several immune and blood cell types. A third panel was collected for lung based on data from Madissoon et al. ^11^ which generated scRNA-seq data using samples undergoing cold preservation (panel name: Madissoon_Lung, **Table S1**). We calculated the average gene expression for each cell type, resulting in 7330 genes in 28 cell types for lung.
9. Five panels for pancreas. The first panel (ArrayExpress ID: E-MTAB-5061) contained scRNA-seq data of human pancreas from healthy individuals and type 2 diabetes patients^12^. We focused on the 7 cell types that each had >=30 cells and discarded the rare cell types reported in the original study: acinar (185 cells), alpha (886), beta (270), delta (114 cells), ductal (386), gamma (197), and PSC (54). In total, there were 13403 genes after filtering those lowly expressed genes. The second panel (GEO ID: GSE81547)^13^ included ~2500 human pancreas cells from eight donors. We used the 6 cell types reported in the original study (acinar: 411, alpha: 998, beta: 348, delta: 83, ductal: 389, and mesenchymal: 53), each containing 9364 – 10519 detected genes (defined as those with non-zero expression). The third panel (GEO ID: GSE81608)^14^ included expression for ~16,000 genes in 4 cell types from non-diabetic and type 2 diabetes donors for human pancreas. The fourth panel (GEO ID: GSE84133)^15^ was collected from a scRNA-seq data including over 12,000 pancreatic cells from both human and mouse. The original study reported 14 cell types identified from the human donors. After excluding immune cells (not unique to pancreas) and cell types with too few cells (<30), we selected ~8000 human pancreatic cells categorized into seven cell types (acinar, alpha, beta, delta, ductal, endothelial, gamma) and 8175 genes, with a minimum of 252 detected genes (endothelial) and a maximum of 2525 detected genes (beta) expressed in each cell type. The fifth panel (GEO ID: GSE85241)^16^ adopted the multiplexed linear amplification (CEL-Seq) protocol and conducted deep sequencing of human pancreas cells from four donors. We collected data for 12461 genes in 8 cell types (acinar: 267; alpha: 852; beta: 465; delta: 199; duct: 278; endothelial: 23; mesenchymal: 97; and pp: 104), with a median of 12400 detected genes per cell type. Note the endothelial cell type had less than 30 cells but to keep it comparable to other pancreas panels, we choose to keep this cell type for the following analyses.
10. Data for spleen and esophagus. The same Madissoon et al. ^11^ study generated scRNA-seq data for spleen and esophagus using samples undergoing cold preservation. We collected these data to prepare panels for these two tissues, respectively (**Table S1**). The same preprocessing steps were conducted as described above.
11. Data for Decidual cells. This dataset contains ~70,000 cells from first-trimester placentas with matched maternal blood and decidual cells^17^. The major cell types of this dataset include different immune and stromal cells.

**Data preprocess**

Although the summary statistics from each GWAS dataset (z-values transformed from SNP p-values) mostly followed the standard normal distribution after study-wise quality control, the gene-based z-values transformed from Magma p-values for each GWAS dataset rarely formed a distribution approximate to the standard normal distribution. Even worse, some GWAS datasets had extremely strong signals in certain genomic regions, such as the APOE loci in Alzheimer’s disease (ALZ), and genes in such regions tended to dominate the results if not normalized appropriately. We used the Box-Cox method to preprocess and transform the gene-based p-values from each GWAS dataset to a distribution approximate to the standard normal distribution (see Methods). Because the Box-Cox method only applies to positive values, we utilized -log10(p) rather than the widely used z-score, to transform gene-based p-values. For each dataset, we examined the lambda values from -2 to 2, with 0.05 as the step. The value that generated a distribution most approximate to the normal distribution would be selected. **Figure 2(A-J)** showed an example using the GWAS dataset for bipolar disorder (BD), where the original data was right skewed. A lambda value of 0.06 was selected and the distribution after transformation was much more alike to the normal distribution. The same Box-Cox transformation procedure was applied to each of the scRNA-seq data sets because the average expression data for each cell type are also heavily skewed.

To integrate the GWAS data and the average expression data for each cell type, we proposed a calibration method to conduct quantile normalization of the two datasets such that they are compatible with each other. In particular, we created a reference dataset following the standard normal distribution where the number of values in the reference dataset is the same as the number of shared genes with both GWAS p-values and non-zero expression in the investigated cell type. The three datasets, i.e., transformed GWAS values, the average expression in a cell type, and the reference dataset, formed a N × 3 matrix and was subsequently analyzed using the quantile normalization. As a result, all three datasets were approximate to the normal distribution and were less skewed or scattered than the input data. scGWAS was then conducted based on the normalized data.

**Methodology design of scGWAS**

*The penalty factor in module score calculation*. We included the penalty factor $sd (m_{g},m_{s})$ in the calculation of module scores to control deviation between GWAS signals and scRNA-seq signals, similar as the regulation factor often used in ridge or lasso regression. Using schizophrenia as an example, which was well-known for its association with several neuron cell types^18^, we tested two ways of module score calculation: (1) without the penalty factor: $m=m_{g}+m_{s}$, and (2) with the factor: $m=m_{g}+m_{s}-sd (m_{g},m_{s})$. As shown in **Figure S1**, without the factor, less modules were found compared when we included the penalty factor and the two types of data drifted away from each other in random cases (red circle in **Figure S1**). Thus, inclusion of the penalty factor can enforce the method to search for modules towards both high GWAS signals and high scRNA-seq signals. In addition, the penalty factor is actually given a weight of 1, i.e., $m=m_{g}+m_{s}-\lambda\times sd (m_{g},m_{s})$, where $\lambda=1$. While $\lambda$ can have other values, it should not be too large because large $\lambda$ values would assign a strong constrain to enforce GWAS signal to be consistent with scRNA-seq signal and our aim is to find modules with combined information from the two data types.

*Inclusion threshold* $r1$ *and exclusion threshold* $r2$. The MEBE method constructs a module by starting with a seed gene and then, the module expands and shrinks until it reaches a stable status. Specifically, the module expands by including the neighbor gene that introduces the highest improvement on the module score until the highest increase fails to satisfy $m^{t+1}>m^{t}\times(1+r1)$. The module also trims non-essential component genes if $m^{t-1}>m^{t}\times(1-r2)$. Thus, $r1$ and $r2$ are related to the final module sizes. Large *r*1 values may result in small modules (hence, missing important nodes) while small *r*1 values may result in large modules (hence, including noisy nodes). In our previous work^19,20^, we have tried different combinations for these two parameters, e.g., (*r*1 = 0.4, *r*2 = 0.2), (*r*1 = 0.2, *r*2 = 0.1), and (*r*1 = 0.1, *r*2 = 0.05), and found that $r1$ and $r2$ values depended on the scale of the input data types and the scale of module scores. In the case to integrate GWAS and scRNA-seq data, we used the random modules to explore candidate values for $r1$ and $r2$ because the random modules represent the null distribution of module scores when the GWAS data and cell type expression data are not related. For each trait and cell type pairs, we collected the random modules and calculated the increasing ratio of module scores by module size being 3 to 9 (note that very few modules could have a size of 10 or larger). That is, $ratio=(m_{k}-m_{k-1})/m_{k-1}$, where $m_{k}$ is the average module score for modules with size *k* and *k* = 4,…,9. As shown in **Figure S2**, when module size increase from 4 to 5, the increase ratio is 12.7% (the vertical line in red); when module size increase from 5 to 6, the ratio is 9.4%; when module size increase from 6 to 7, the ratio is 6.9%; when module size increase from 7 to 8, the ratio is 5.6%; and when module size increase from 8 to 9, the ratio is 3.5%. Thus, $r1$= 0.1 would result in modules mostly at size 7 and few modules can keep expanding to a size of 8 or larger. This is consistent with our general understanding about GWAS data and scRNA-seq data because it is unlikely to have a large module where the component genes have both significant GAWS p-values and high cell type expression. With the value of $r1$ being 0.1, we set $r2$ to be half the value of $r1$. Thus, with our settings of $r1$and $r2$, every time when the module includes one more node, the module score increases by at least 10%; and every time the module excludes one node, the module score decreases by at most 5%.

*Virtual search procedure*. In module search tasks, estimation of the significance of the resultant modules have been proven hard because the null distribution of the module scores is unknown^21-23^. Many previous works, including some from our own group^21,23^, used the permutation strategy which permuted the node weight among the nodes in the graph, recalculated the module scores in each permutation, and calculated a z-score based on the permutation process for each module (termed permutation-based z-score). However, such a strategy failed to account for the size effect and modules with more nodes still had larger permutation z-scores than modules with less nodes (**Figure S3**). Using the virtual search procedure, we generate random modules in each permutation, group the random modules by their node sizes, and then calculate module scores per group. As shown in **Figure S3**, the effects of module sizes are in general much reduced using the virtual search procedure. Thus, this new strategy can effectively normalize the module scores.

*Selection of average gene expression per cell type*. We used the average gene expression to represent the transcriptome of each cell type. However, any forms of measurement of the cell-type expression can be used as input to the method. Some representative examples included the specificity measurement (*t*-score or *z*-score)^24^, the variance of each gene in a cell type, or the score by comparing “one versus other” cell types as implemented in Seurat^25^, among others. Users can freely select such measurement and scGWAS seeks for modules where the component genes are simultaneously enriched with both the GWAS signals and the cell type measurements.

*Comparison with FUMA*. FUMA uses MAGMA to test cell-type specificity of GWAS summary statistics. This is similar to the first aim of scGWAS. We thus used schizophrenia as an example to compare the cell types identified by scGWAS and by FUMA. As shown in **Figure S4**, scGWAS recovered all excitatory neurons and inhibitory neurons that were reported by FUMA and reported all the cell types from fetal brain as insignificant, which was also consistent with FUMA results. These results indicated that scGWAS could achieve comparable results as FUMA in terms of identifying trait-associated cell types. However, scGWAS can also identify the subnetworks to show how each component gene mediated the cell-type association.

**References**

1. 10x Genomics website. <https://support.10xgenomics.com/single-cell-gene-expression/datasets>.

2. Wang, D. *et al.* Comprehensive functional genomic resource and integrative model for the human brain. *Science* **362**(2018).

3. Darmanis, S. *et al.* A survey of human brain transcriptome diversity at the single cell level. *Proc Natl Acad Sci U S A* **112**, 7285-90 (2015).

4. Lake, B.B. *et al.* Neuronal subtypes and diversity revealed by single-nucleus RNA sequencing of the human brain. *Science* **352**, 1586-90 (2016).

5. Lake, B.B. *et al.* Integrative single-cell analysis of transcriptional and epigenetic states in the human adult brain. *Nat Biotechnol* **36**, 70-80 (2018).

6. Zeisel, A. *et al.* Brain structure. Cell types in the mouse cortex and hippocampus revealed by single-cell RNA-seq. *Science* **347**, 1138-42 (2015).

7. Saunders, A. *et al.* Molecular diversity and specializations among the cells of the adult mouse brain. *Cell* **174**, 1015-1030 e16 (2018).

8. Cui, Y. *et al.* Single-cell transcriptome analysis maps the developmental track of the human heart. *Cell Rep* **26**, 1934-1950 e5 (2019).

9. MacParland, S.A. *et al.* Single cell RNA sequencing of human liver reveals distinct intrahepatic macrophage populations. *Nat Commun* **9**, 4383 (2018).

10. Travaglini, K.J. *et al.* A molecular cell atlas of the human lung from single-cell RNA sequencing. *Nature* **587**, 619-625 (2020).

11. Madissoon, E. *et al.* scRNA-seq assessment of the human lung, spleen, and esophagus tissue stability after cold preservation. *Genome Biol* **21**, 1 (2019).

12. Segerstolpe, A. *et al.* Single-Cell Transcriptome Profiling of Human Pancreatic Islets in Health and Type 2 Diabetes. *Cell Metab* **24**, 593-607 (2016).

13. Enge, M. *et al.* Single-cell analysis of human pancreas reveals transcriptional signatures of aging and somatic mutation patterns. *Cell* **171**, 321-330 e14 (2017).

14. Xin, Y. *et al.* RNA Sequencing of Single Human Islet Cells Reveals Type 2 Diabetes Genes. *Cell Metab* **24**, 608-615 (2016).

15. Baron, M. *et al.* A single-cell transcriptomic map of the human and mouse pancreas reveals inter- and intra-cell population structure. *Cell Syst* **3**, 346-360 e4 (2016).

16. Muraro, M.J. *et al.* A single-cell transcriptome atlas of the human pancreas. *Cell Syst* **3**, 385-394 e3 (2016).

17. Vento-Tormo, R. *et al.* Single-cell reconstruction of the early maternal-fetal interface in humans. *Nature* **563**, 347-353 (2018).

18. Skene, N.G. *et al.* Genetic identification of brain cell types underlying schizophrenia. *Nat Genet* **50**, 825-833 (2018).

19. Jia, P., Pei, G. & Zhao, Z. CNet: a multi-omics approach to detecting clinically associated, combinatory genomic signatures. *Bioinformatics* **35**, 5207-5215 (2019).

20. Jia, P., Manuel, A.M., Fernandes, B.S., Dai, Y. & Zhao, Z. Distinct effect of prenatal and postnatal brain expression across 20 brain disorders and anthropometric social traits: a systematic study of spatiotemporal modularity. *Brief Bioinform* **22**(2021).

21. Jia, P., Zheng, S., Long, J., Zheng, W. & Zhao, Z. dmGWAS: dense module searching for genome-wide association studies in protein-protein interaction networks. *Bioinformatics* **27**, 95-102 (2011).

22. Reyna, M.A., Chitra, U., Elyanow, R. & Raphael, B.J. NetMix: A Network-Structured Mixture Model for Reduced-Bias Estimation of Altered Subnetworks. *J Comput Biol* **28**, 469-484 (2021).

23. Jia, P. *et al.* Network-assisted investigation of combined causal signals from genome-wide association studies in schizophrenia. *PLoS Comput Biol* **8**, e1002587 (2012).

24. Dai, Y. *et al.* CSEA-DB: an omnibus for human complex trait and cell type associations. *Nucleic Acids Res* **49**, D862-D870 (2021).

25. Satija, R., Farrell, J.A., Gennert, D., Schier, A.F. & Regev, A. Spatial reconstruction of single-cell gene expression data. *Nat Biotechnol* **33**, 495-502 (2015).

**Table S1**. Summary of collected scRNA-seq datasets

| Tissue | Panel name | # cells (raw) | # cell types  (raw) | # cell types  (≥30) | # genes | Download data | Transformation | URL or reference |
| --- | --- | --- | --- | --- | --- | --- | --- | --- |
| Blood | PBMC10k | 94655 | 10 | 10 | 5655 | UMI | Log2(CPM+1) | ^1^ |
| Brain | DER20 | 4250 | 35 | 28 | 14306 | TPM | Log2(TPM+1) | ^2-4^ |
|  | DER22 | 27412 | 25 | 25 | 14025 | UMI | Log2(CPM+1) | ^2,5^ |
|  | Zeisel | 160,796 | 39 | 39# | 15827 | Expression values per cluster | Log2(value+1) | https://www.ncbi.nlm.nih.gov/sra/SRP135960^6^ |
|  | Saunders | 690,000 | 88 | 88# | 14459 | Count | Log2(CPM+1) | http://dropviz.org/^7^ |
| Heart (fetal) | Heart | 4949 | 9 | 9* | 13095 | UMI | Log2(CPM+1) | GSE106118^8^ |
| Liver | Liver | 8445 | 20 | 20 | 6717 | Count | Log2(CPM+1) | ^9^ |
| Lung | LungSS2 | 9409 | 44 | 33 | 11396 | Count | Log2(CPM+1) | ^10^ |
|  | Lung10x | 65662 | 57 | 53 | 8763 | UMI | Log2(CPM+1) | ^10^ |
|  | Madissoon_Lung | 57020 | 28 | 28 | 7330 | UMI | Log2(CPM+1) | ^11^ |
| Pancreas | E-MTAB-5061 | 3514 | 7 | 7 | 13403 | Count | Log2(CPM+1) | ^12^ |
|  | GSE81547 | 2282 | 6 | 6 | 10524 | Count | Log2(CPM+1) | ^13^ |
|  | GSE81608 | 1600 | 4 | 4 | 16293 | RPKM | Log2(RPKM+1) | ^14^ |
|  | GSE84133 | 7994 | 7 | 7 | 8175 | Count | Log2(CPM+1) | ^15^ |
|  | GSE85241 | 2119 | 8 | 8 | 12472 | Normalized counts | Log2(CPM+1) | ^16^ |
| Spleen | Spleen | 94257 | 30 | 30 | 4497 | UMI | Log2(CPM+1) | ^11^ |
| Esophagus | Esophagus | 87947 | 19 | 19 | 7288 | UMI | Log2(CPM+1) | ^11^ |
| Decidua | Decidua | 5591 | 31 | 23 | 33109 | UMI | Log2(CPM+1) | ^17^ |

**
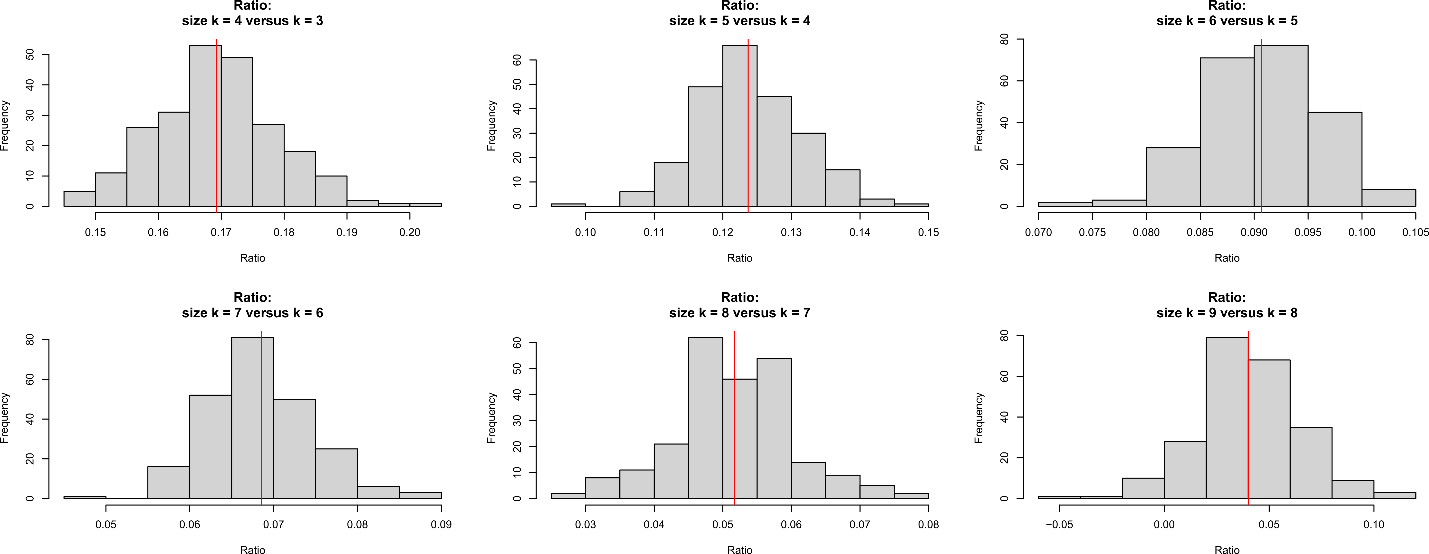
**

**Figure S1. Distribution of module score increasing ratio with different module sizes**. The red line indicates the mean ratio for each specific module size.


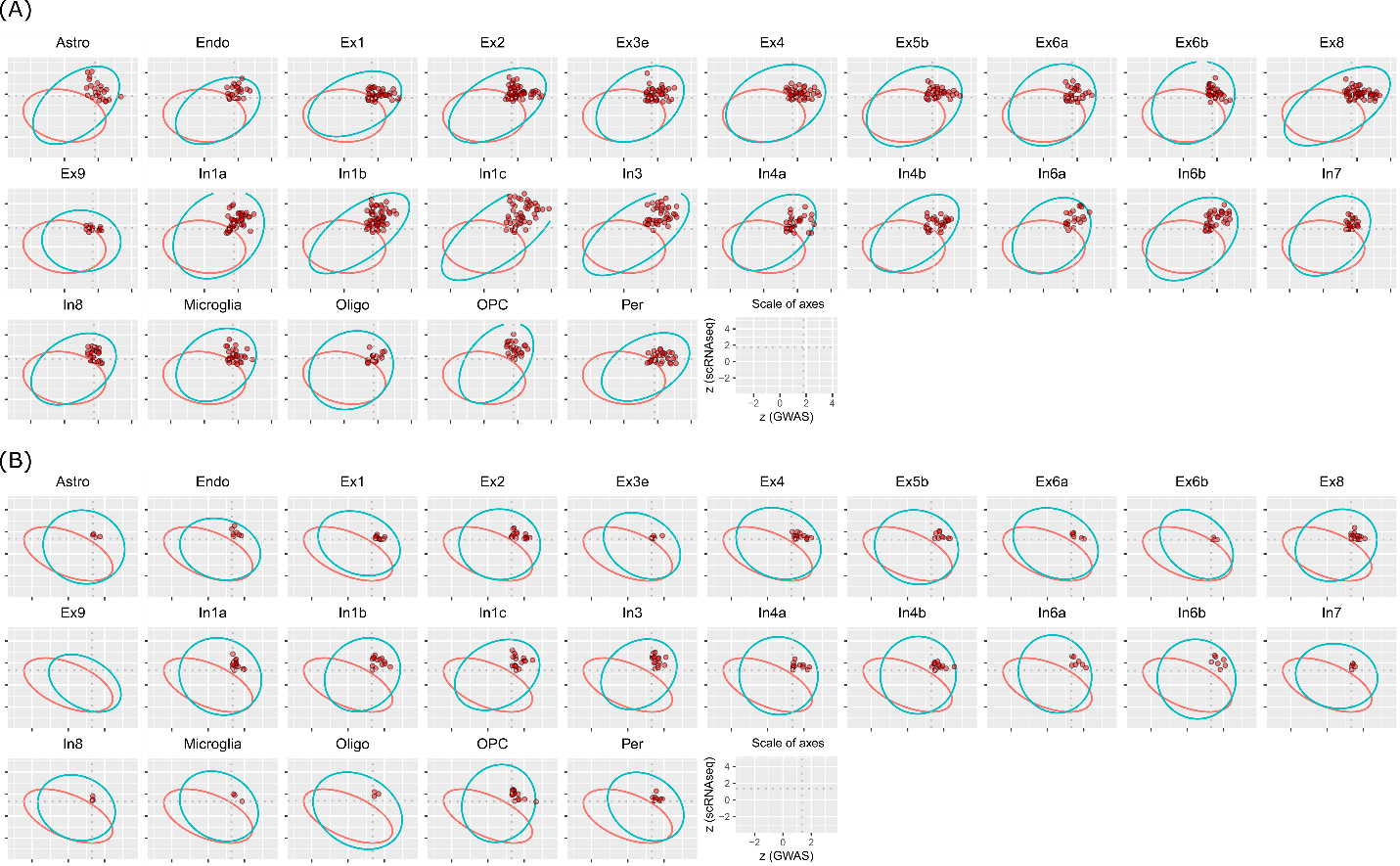


**Figure S2. Comparison of different methods to calculate module score**. (A-B) The module score distribution for schizophrenia using the DER22 panel and the module score calculation with penalty (A, above) and without penalty (B, bottom). In both panels, the last plot shows normalized module score from scRNA-seq (y-axis) versus the normalized module score from GWAS (x-axis). In each panel, the red circle indicates the 95% confidence interval (CI) estimated using the random modules and the blue circle indicates the 95% CI estimated using the real modules. Significant modules are highlighted in red while all other modules, including non-significant modules from real data and all random modules from the virtual runs, are not plotted for simplicity.


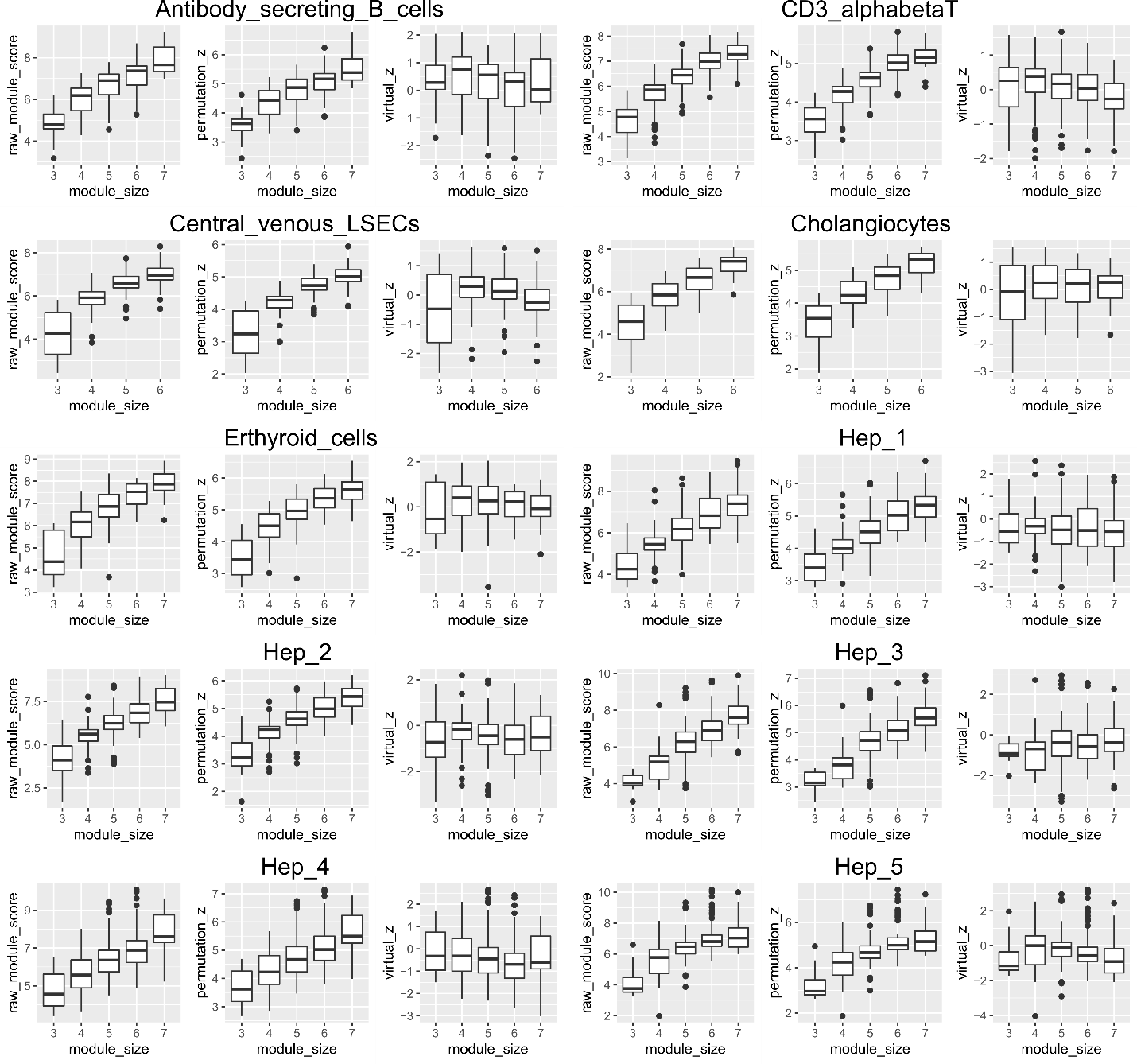


**Figure S3.** **Comparison of different normalization methods for module scores**. Using 10 cell types from the liver panel as an example. For each cell type, we show three types of module score distribution: the raw module score, permutation-based z-score, and the z-score based on size-matched random modules from the virtual search process.


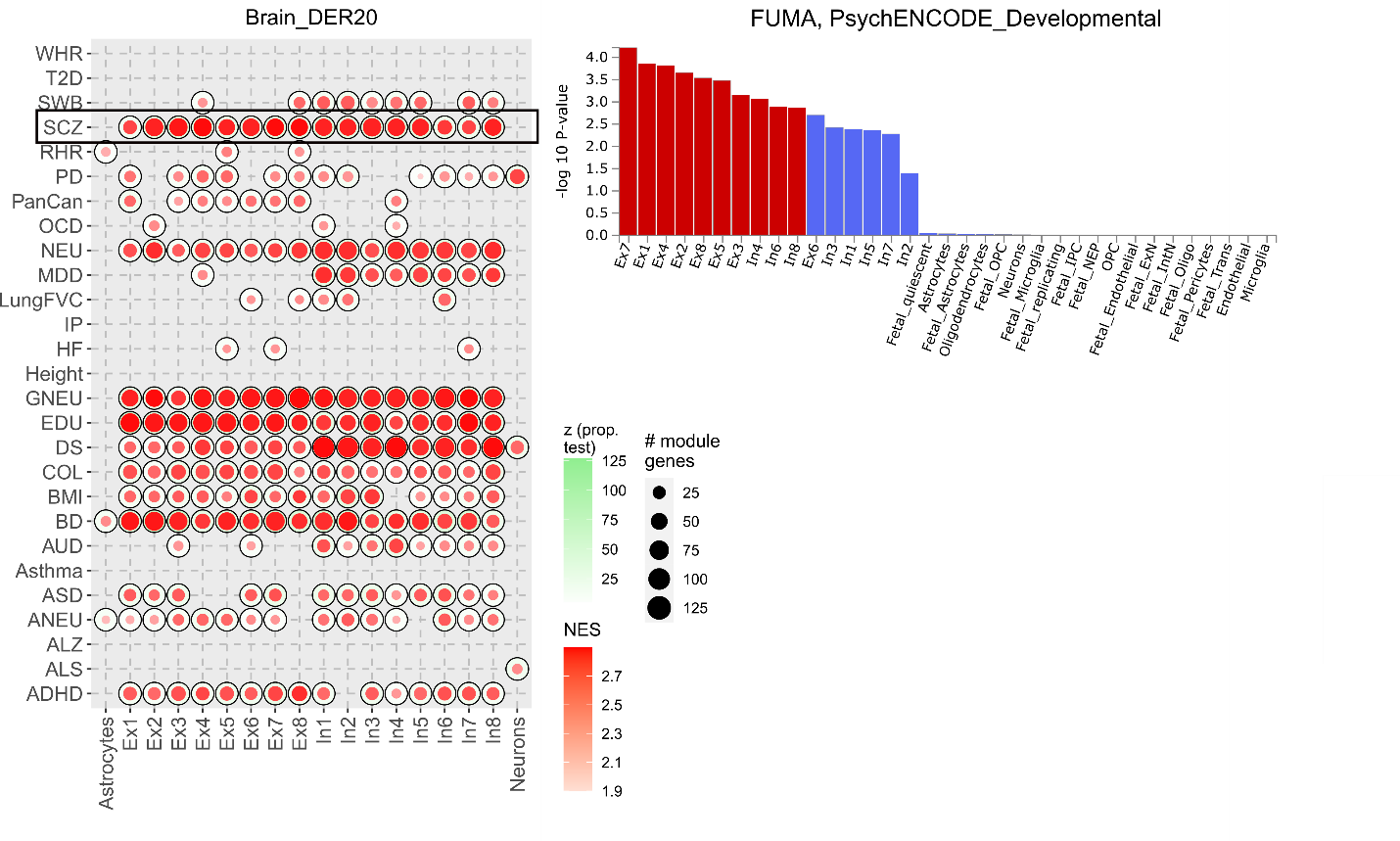


**Figure S4. Comparison of results between scGWAS and FUMA**. (A) Trait and cell type association in the PsychENCODE developmental dataset which is also referred to as DER20 in our work. (B) Association results from FUMA using the same PsychENCODE developmental dataset.


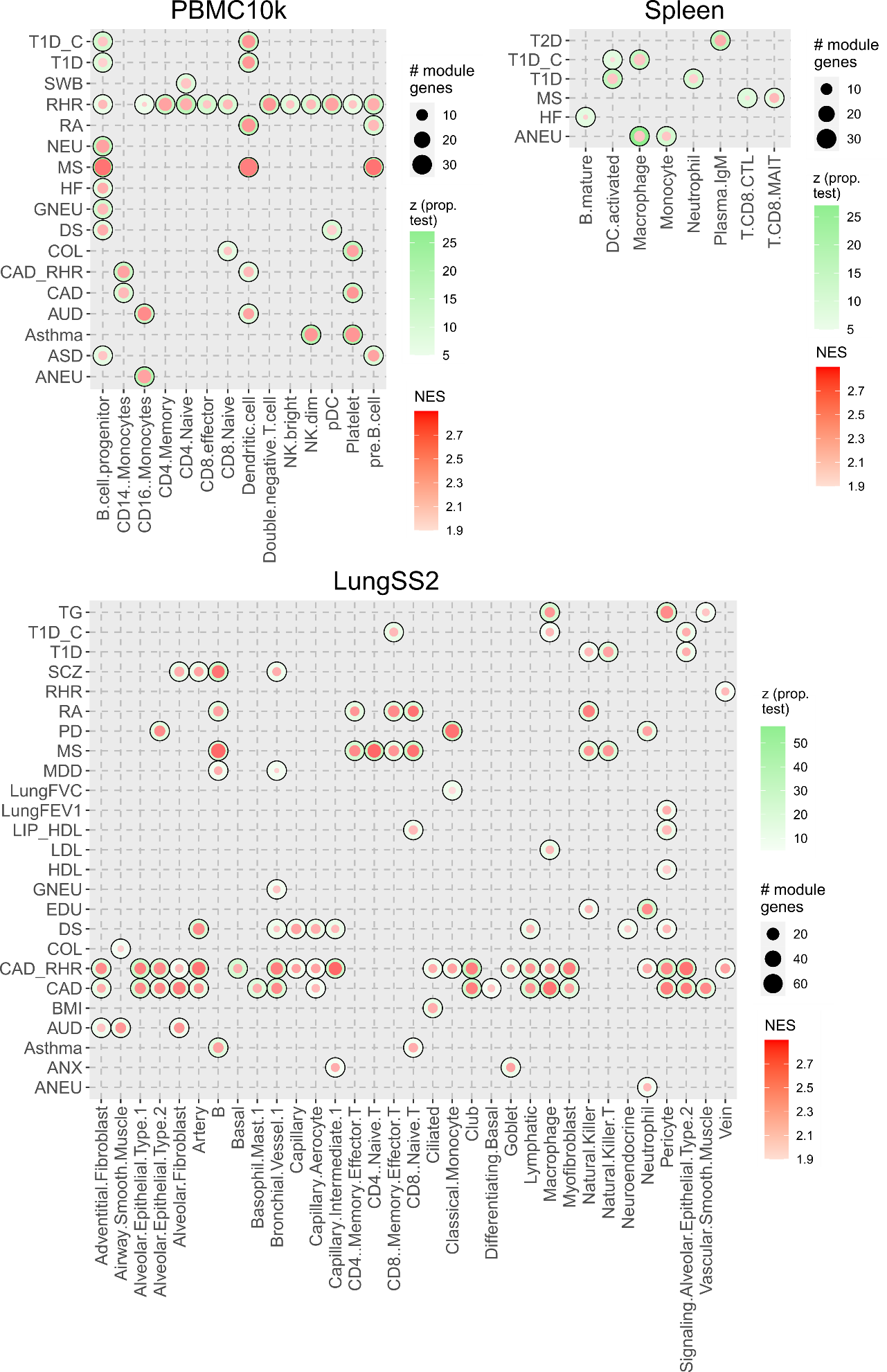


**Figure S5.** Trait and cell type association in the PBMC, spleen, and LungSS2 panels. For each trait and cell type association, there are three types of information included: the outside circle indicates the association level (the intensity of green is proportional to the z-score from the proportional test), the inside circle indicates the significance of module genes (the intensity of red is proportional to the NES value of module genes) and the size indicates the member of module genes.


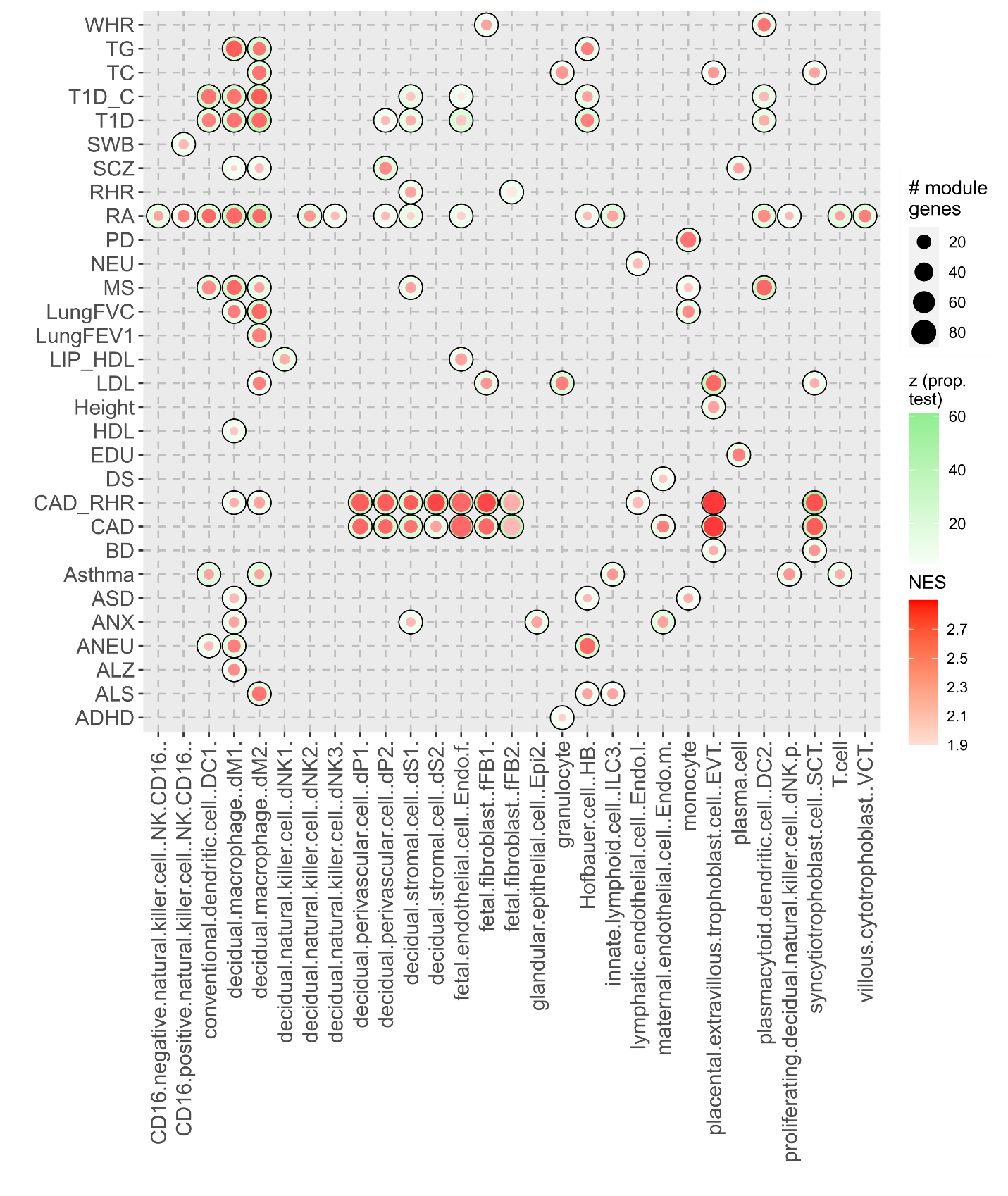


**Figure S6.** Trait and cell type association in the decidua panel. Figure legend is the same as in Figure S4.


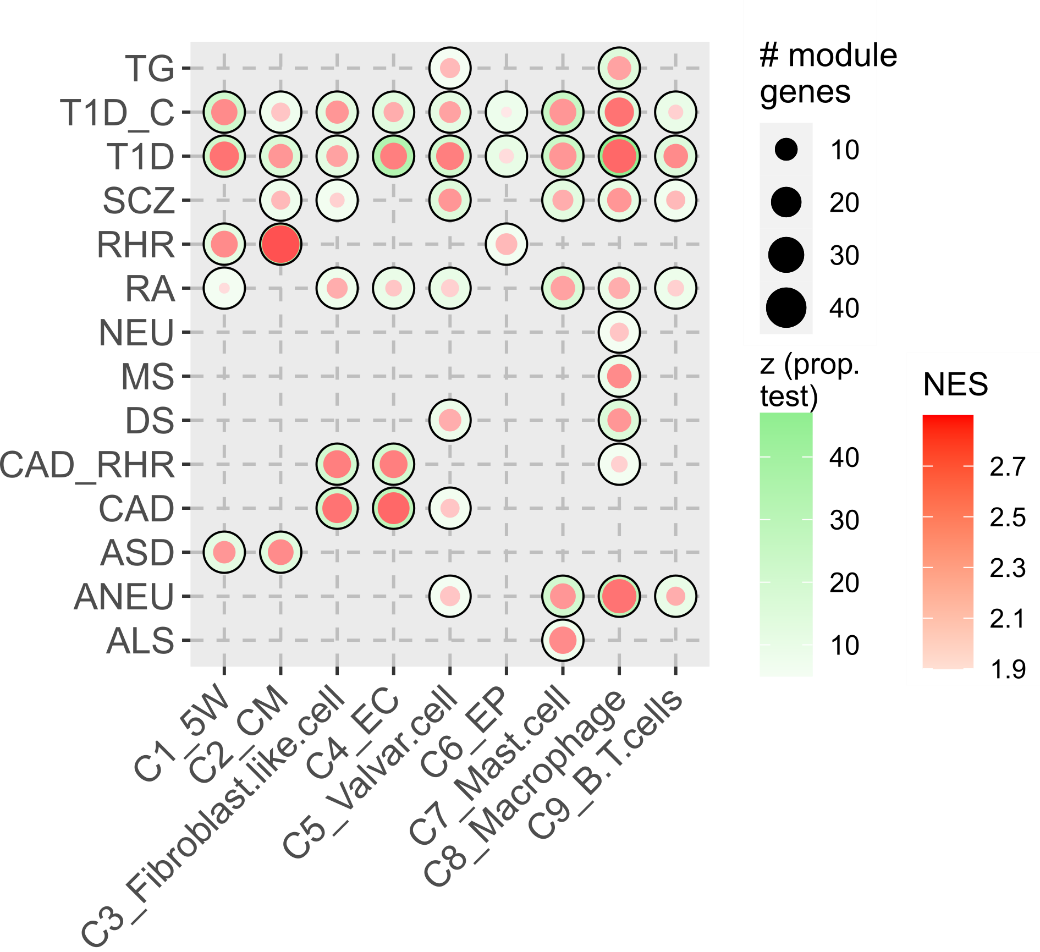


**Figure S7.** Trait and cell type association in the heart panel. Figure legend is the same as in Figure S4.
