# Supplementary material for: scGWAS: landscape of trait-cell type associations by integrating single-cell transcriptomics-wide and genome-wide association studies
Source: Genome Biol. 2022 Oct 17;23:220. doi: 10.1186/s13059-022-02785-w (PMC9575201; doi:10.1186/s13059-022-02785-w)
Supplement: Supplementary file 2 — Additional file 2: Review history. [file 13059_2022_2785_MOESM2_ESM.docx]

Review History

**First round of review**

**Reviewer 1**

**Were you able to assess all statistics in the manuscript, including the appropriateness of statistical tests used?** Yes, and I have assessed the statistics in my report.

**Comments to author:**

In this manuscript, Jia and colleagues present scGWAS that harnesses the power of rapidly accumulating single-cell RNA sequencing (scRNA-seq) data for more comprehensive genetic dissection of diseases and traits. Specifically, scGWAS can be used for two purposes: (1) to infer the most relevant cell types; and (2) to construct disease-specific cellular modules via network inference. The manuscript is well organized and clearly written. The authors have made serious efforts in applying their scGWAS method to 40 GWAS studies across a spectrum of complex diseases and traits, while simultaneously leveraging scRNA-seq data from 18 datasets involving a total of over one million cells. As single cell omics is rapidly evolving as the main approaches in biological and biomedical research, and numerous GWA studies have been published and more in the near future, scGWAS will be a powerful and handy tool for the research community, benefiting many investigators in this post-GWAS era. The method is novel and timely. I am very enthusiastic with the work and comments below are meant for further improving the presentation or purely for my own curiosity. I have only one major comment: the GitHub should be substantially improved to allow other investigators to use scGWAS for their own study in a reproducible manner.

I also have a few minor comments regarding technical details (in no particular order).

* Have the authors calculated module score for GWAS and scRNA-seq separately? It would be informative to compare the module identified only by GWAS/scRNA-seq.

* The authors used average gene expression profile from scRNA-seq data. How does sparsity affect this? Might miss genes by just taking the mean. Maybe try some "robust mean"/"robust median" controlling sparsity.

* How likely would the reference network (background network) affect the performance of scGWAS?

* For the Module score section, what are N and ν? It seems that ν is the number of nodes and N is the number of modules. The authors should explicitly explain the notations.

* Figures can be further polished. For example, Figure 3B and Figure 7B need a color scale to specify the value for the colors.

**Reviewer 2**

**Were you able to assess all statistics in the manuscript, including the appropriateness of statistical tests used?** Yes, and I have assessed the statistics in my report.

**Comments to author:**

In this paper Jia et al. present scGWAS a computational method to infer relevant disease associated cell types and gene modules based on preprocessed scRNA-seq data with cell type annotations and GWAS summary statistics.

Despite the proposed analysis is interesting the idea is not novel and the manuscript has several assumptions, empirical procedures and ad-hoc parameters that will make its general applicability not trivial. The exposition and language can be also improved.

1) The gene score based on MAGMA is based only on proximal region for each gene (i.e. 50 kb upstream and 35 kb downstream), however as stated in the manuscript many GWAS are in non-coding regions, and it is well known that many enhancers can be located up to 1M from their target genes. Also the SNPs to genes assignment is just based on proximity potentially introducing false positive signals.

2) Despite the authors suggest that single-cell information is leveraged by their method, in reality only cell type averages are used based on already annotated datasets and co-expression between genes is totally ignored to define the significant modules. So, the method in principle is similar to many other methods based on bulk RNA expression and GWAS summary statistics(see point 8). Based on this I found this statement "In summary, scGWAS is a powerful tool for deeply exploring the genetic basis of complex diseases at the single cell level." and similar statements in the manuscript misleading.

3) It is not reported how cell types were defined based on the different scRNA-seq datasets. Given that different studies may have used very different procedures to define cell types it is important to discuss this and to provide guidelines for the users interested in applying the scGWAS tool on a new dataset without annotations.

4) I found the attempt to map GWAS gene scores and scRNA-seq scores to normal distribution very artificial and ad hoc. For example, as also observed by the authors scRNA-seq data after their proposed procedures is still right skewed. Therefore, additional corrections are necessary downstream, and the entire procedure is composed of a series of ad-hoc and not well justified transformations.

5) Several thresholds are necessary to define the modules (e.g. r1 and r2) however it is not discussed in detail how these thresholds should be optimally adjusted for a new dataset, but only empirical values are provided in the supplementary material.

6) Virtual module search: this procedure is based on permutations of existing weights. The author report to have at least 100 for each module size, however they then assume a normal distribution z-scoring the resulting module scores. However, this assumption was not tested and with only 100 permutations it is not clear how they can set the threshold to pm<0.05. Also there are some typos "Virtue" instead of "Virtual" in the main text.

7) Assessment of combined module: the authors used GSEA and in particular NES scores to measure "overall significance" line 51 page 8. However later they state that this score is only used for "presentation purpose". Also, I don't understand the final sentence "Thus there is no data circulation issue". This sentence is also repeated in the discussion

8) Previous relevant work is not acknowledged, and the manuscript lacks a reasonable comparison with existing methods: e.g. https://www.ncbi.nlm.nih.gov/pmc/articles/PMC5896795/ (see in particular Figure 1 for the proposed workflow for RNA+GWAS summary statistics).

https://www.ncbi.nlm.nih.gov/pmc/articles/PMC5673624/

https://www.frontiersin.org/articles/10.3389/fgene.2021.637322/full

https://genomemedicine.biomedcentral.com/articles/10.1186/s13073-022-01021-1

and https://www.nature.com/articles/s41467-019-11181-1 .

Please compare scGWAS with with LD score regression, RolyPoly and FUMA.

9) The software package is poorly documented and only a succinct example is provided on GitHub. The documentation needs to be expanded and the expected outputs described. Also, to ensure reproducibility it is important to show how the package was used to obtain the results presented in the manuscript and share the additional R scripts used.

**Authors Response**

**Response to the comments of the reviewers**

Manuscript ID: GBIO-D-22-00281

Title: Landscape of trait-cell type associations by integrating single-cell transcriptomics-wide and genome-wide association studies (scGWAS)

We appreciate very much the valuable comments of the reviewers. We have spent substantial time and effort on revision. Each of these comments has been taken into account in the revised manuscript or is discussed below. Specifically, we reprocessed the reference network data to deal with the SNP-gene mapping problem to reduce potential inflation raised by interacting genes which are closely located in the genome. We also applied the Box-Cox transformation to the scRNA-seq data to ensure that both GWAS and scRNA-seq data follow the normal distribution (Reviewer #2). In addition, we increased the number of virtual runs to generate a good number of random modules (Reviewer #2) and to ensure the stability and reproducibility of the resultant significant modules. We tried our best to compare our scGWAS with peer methods, and to show the unique features of our methods (Reviewer #2). Finally, we enhanced the documentation in GitHub by providing the package, preprocessing codes, post-analysis codes, as well as a work-through example (Reviewers #1 and #2). These additional works indicate our results are reliable. We feel our manuscript has been substantially improved. The following is an outline of our point-to-point responses to reviewers’ major criticisms.

**Reviewer #1:**

*In this manuscript, Jia and colleagues present scGWAS that harnesses the power of rapidly accumulating single-cell RNA sequencing (scRNA-seq) data for more comprehensive genetic dissection of diseases and traits. Specifically, scGWAS can be used for two purposes: (1) to infer the most relevant cell types; and (2) to construct disease-specific cellular modules via network inference. The manuscript is well organized and clearly written. The authors have made serious efforts in applying their scGWAS method to 40 GWAS studies across a spectrum of complex diseases and traits, while simultaneously leveraging scRNA-seq data from 18 datasets involving a total of over one million cells. As single cell omics is rapidly evolving as the main approaches in biological and biomedical research, and numerous GWA studies have been published and more in the near future, scGWAS will be a powerful and handy tool for the research community, benefiting many investigators in this post-GWAS era. The method is novel and timely. I am very enthusiastic with the work and comments below are meant for further improving the presentation or purely for my own curiosity. I have only one major comment: the GitHub should be substantially improved to allow other investigators to use scGWAS for their own study in a reproducible manner.* 

**Response**: We thank the reviewer’s positive comments.

*I also have a few minor comments regarding technical details (in no particular order).*

**       Have the authors calculated module score for GWAS and scRNA-seq separately? It would be informative to compare the module identified only by GWAS/scRNA-seq.*

**Response**: We thank the reviewer’s suggestion. In response to this suggestion, we calculated modules by using only GWAS data or using only scRNA-seq data. We implemented the same virtual search strategy to generate at least 1000 random modules for each module size to construct the null distribution of module scores. We defined significant modules as those with $p_{m}=\frac{\#\{z(\pi)>z_{m}\}}{\#\pi}<0.05$, where $\pi$ is the collection of all random modules. By using GWAS data only, we identified 40 sets of module genes for the 40 traits, respectively. By using scRNA-seq data only, we identified significant modules for each cell type in each panel. Below, we demonstrate the comparison in two aspects: the overall functional impact of module genes and the overlap of module genes identified by either strategy (scGWAS or GWAS only).

By using pLI, ClinVar, and OMIM annotations, we found that overall, the module genes identified by scGWAS tended to be more functionally important than the module genes identified by using GWAS only (the average OR for pLI: 2.38 by scGWAS compared to 1.84 by GWAS only; 1.79 versus 1.64 for ClinVar; and 1.96 versus 1.78 for OMIM). As for module genes identified by scGWAS and module genes identified by scRNA-seq only, they were comparable to each other: the average OR for pLI: 2.38 by scGWAS compared to 1.67 by scRNA-seq only; 1.79 versus 2.03 for ClinVar; and 1.96 versus 1.89 for OMIM. The results are shown in Figure 1 below. Note that the scRNA-seq-only genes (red dots in Figure 1 below) are generally more functionally important. However, we did not pursue these genes further, because these genes are more tissue- or cell type-related, rather than disease-related. Therefore, we focus on the comparison between GWAS only and scGWAS genes.

**Figure 1.** Comparison of module genes using pLI, ClinVar, and OMIM annotations. For each panel (except the third row for “GWAS_only”), we showed the forest plot using four sets using the mean OR with the 25%-75% range. The four sets of genes are the scGWAS-identified module gene set, the GWAS-top set, the scRNA-seq_top set, and the scRNA-seq_only set. The GWAS-top set of genes was constructed by using the top-scored genes ordered by Magma gene-based score with the same size of module genes as selected by the scGWAS method. Similarly, the scRNA-seq_top set of genes was constructed by using the top scored genes ordered by the average cell type expression with the matched size. The third row, i.e., the GWAS_only set, contained 40 sets of genes for each of the 40 traits, where genes in each set were identified by applying the same scGWAS procedure but using only Magma gene-based score to calculate the module score. Similarly, the scRNA-seq_only set of genes were identified by using only the average cell type expression to calculate the module score. For scRNA-seq_only genes, they were identified for each cell type in each panel and thus, for each panel, the red dot and line represent gene sets for each cell type. All: module gene sets from all the trait and cell-type associations. All2: module gene sets with ≥ 20 genes.

For all 40 traits investigated, an average of 44.0% (range: 10.6% to 80.0%) of the GWAS-only genes were also reported by scGWAS in different cell types. We used the Alcohol Use Disorder (AUD) and five well-studied psychiatric disorders (ADHD: Attention Deficit Hyperactivity Disorder; ASD: Autism; BD: Bipolar Disorder; MDD: Major Depressive Disorder; SCZ: Schizophrenia) to investigate the overlap between GWAS-only genes and scGWAS genes in individual cell types. We limited the comparison to the DER20 (PsychENCODE development) and DER22 (PsychENCODE adult) panels because these two panels are from brain and have been widely used to study the association between brain cell types and psychiatric disorders in the previous studies. Figure 2 below shows the overlapping genes for each individual cell type for the 6 traits. For the DER20 panel, across all 35 cell types, up to 36% of scGWAS genes were also covered by GWAS_only genes (top panel with red dots) and up to 38% of GWAS_only genes were covered by scGWAS genes in different cell types (bottom panel with blue dots). When using DER22 panel, up to 26% of scGWAS genes were covered by GWAS_only genes and up to 37% of GWAS_only genes were covered by scGWAS genes in different cell types.

**Figure 2.** Distribution of shared genes identified by using GWAS only or by using both GWAS and scRNA-seq data. For either DER20 or DER22 panel, the top figure (red dots) indicates the proportion of shared genes compared to the genes identified by scGWAS; the bottom figure (blue dots) indicates the proportion of shared genes compared to the genes identified by using GWAS only.

In summary, scGWAS can recover nearly half of the genes reported by using GWAS only but the genes reported by scGWAS tended to be more functionally important, partially attributed to the introduction of cell-type expression profiles. In addition, in different cell types, the reported genes tended to be diverse and were related to the activated processes in the specific cell types. Thus, in addition to the strategy using only GWAS data, scGWAS provided alternative and complementary lists of genes to understand the disease etiology. These results have been added in the revised manuscript.

**       The authors used average gene expression profile from scRNA-seq data. How does sparsity affect this? Might miss genes by just taking the mean. Maybe try some "robust mean"/"robust median" controlling sparsity.*

**Response**: We thank the reviewer’s suggestion. We agree that sparsity is a severe problem in scRNA-seq data. We used average gene expression per cell type and discarded genes whose expression was 0 in all cells in a specific cell type. This strategy was previously employed by RolyPoly (Calderon D et al., 2017. Inferring Relevant Cell Types for Complex Traits by Using Single-Cell Gene Expression. AJHG 10.1016/j.ajhg.2017.09.009). The problem with this strategy, which is also the direct result of sparsity, is that the resultant expression profile per cell type is highly skewed with a long tail. Many lowly expressed genes with large sparsity formed the skewed distribution. During revision, we introduced the Box-Cox transformation to force the original distribution to approximate the normal distribution. This transformation does not change the order of genes. While the normal distribution is symmetric (two-sided), we only test the right side where genes with high average expression are located. The lowly expressed genes due to large sparsity thus have less impact on the significant modules.

In addition, we have implemented scGWAS as a user-friendly Java package. In this package, it allows users to define their own way of cell expression profile, which can be any forms of robust mean or robust median.

In the revised manuscript, the Discussion section, page 36, we added:

“*In addition, other forms of measurement can be chosen, e.g., gene expression variance (as highly variably expressed genes are often used to conduct classification analysis), other forms of mean or median, cell-type specificity (as implemented in our previous work CSEA-DB), among others*.”

**       How likely would the reference network (background network) affect the performance of scGWAS?*

**Response**: We thank the reviewer to raise this question. scGWAS makes no assumption about the reference network. Any type of gene-gene relationship can be used to serve as the reference network, as long as the network represents the majority of human genes. In our work, we used a network with a wide variety of relationships, including catalysis, chemical effect, regulation of expression or phosphorylation, react, interacts-with, among others. Users can provide their own definition of reference networks, e.g., co-expression, protein-protein interaction, genetic regulation, and so on. Accordingly, the interpretation of the scGWAS results will be adjusted by such reference networks.

In the revised manuscript, the Discussion section, page 37, the second paragraph, we added:

“*Second, scGWAS makes no assumption about the reference network. Any type of gene-gene relationship can be used to serve as the reference network, e.g., co-expression, protein-protein interaction, genetic regulation, and so on. Accordingly, the interpretation of the scGWAS results will be adjusted by such reference networks*.”

**       For the Module score section, what are N and ν? It seems that ν is the number of nodes and N is the number of modules. The authors should explicitly explain the notations.*

**Response**: We apologize for the confusion. We used the letter v in a lower case to represent a node. When v has a subscript, it denotes the weight of the node, i.e., v_g_ for the node’s weight calculated based on GWAS data and v_s_ for the node’s weight calculated based on scRNA-seq data. We used the format |v| to represent the number of nodes in a module. *N* is the total number of nodes. In the revised manuscript, we have rewritten the related text to clarify these terms.

**       Figures can be further polished. For example, Figure 3B and Figure 7B need a color scale to specify the value for the colors.*

**Response**: We apologize for the confusion. In the revised manuscript, we have added a color scale for both figures and also carefully checked all figures for potentially missed legend.

*Reviewer #2: In this paper Jia et al. present scGWAS a computational method to infer relevant disease associated cell types and gene modules based on preprocessed scRNA-seq data with cell type annotations and GWAS summary statistics.

Despite the proposed analysis is interesting the idea is not novel and the manuscript has several assumptions, empirical procedures and ad-hoc parameters that will make its general applicability not trivial. The exposition and language can be also improved.*

**Response:** We thank the reviewer for carefully reading our manuscript and valuable comments on our method. In revision, we asked a paid professional English editing service to enhance English writing. We acknowledged this service in the Acknowledgement section.

*1)      The gene score based on MAGMA is based only on proximal region for each gene (i.e. 50 kb upstream and 35 kb downstream), however as stated in the manuscript many GWAS are in non-coding regions, and it is well known that many enhancers can be located up to 1M from their target genes. Also the SNPs to genes assignment is just based on proximity potentially introducing false positive signals.*

**Response**: We thank the reviewer for this critical point. We agree that our current way of mapping SNPs to genes using 50kb/35kb might miss some SNPs in important regulatory regions to genes. However, using proximal region has been the common practice and the most efficient and practical ways to map SNPs to genes, including many of others (including FUMA) and our prior studies. The rationale underlying this strategy is the linkage disequilibrium (LD). SNPs within 100 kb or similar ranges typically share the same LD; that is also how to infer the potential causal signals from association analysis.

Although regulatory SNPs may be located far away, a large proportion of regulatory variants are located close to their target genes, which has been demonstrated in many QTL studies. If we expanded the region to 1M or even longer, it would cause many false association signals. If we only used the know regulatory SNPs that can link to their genes, we would have very limited number of SNPs linking to genes, and even so, such regulatory SNPs may not really function in the specific cells or cell types. When these factors are considered, we consider using proximal region is a more reasonable strategy, which can capture the major signals and is sensitive enough for our current work. In our previous work, we have successfully used MAGMA to infer tissues and cell-types associated with diseases.

In response to this concern, we also leave the option open for users to provide their own way of mapping SNPs to genes and calculating gene-based scores based on any regulatory regions, including enhancers. scGWAS assumes the gene-based scores from GWAS data have biologically important meanings and searches for modules where GWAS-implied genes and cell-type genes are concordantly enriched. In the revised manuscript, we added a sentence to alert users when using MAGMA to map SNPs to genes (page 23):

“*Of note, mapping SNPs only in proximal regions to genes may miss important regulatory regions which can be located up to 1Mb away from the gene.*”

*2)      Despite the authors suggest that single-cell information is leveraged by their method, in reality only cell type averages are used based on already annotated datasets and co-expression between genes is totally ignored to define the significant modules. So, the method in principle is similar to many other methods based on bulk RNA expression and GWAS summary statistics(see point 8). Based on this I found this statement "In summary, scGWAS is a powerful tool for deeply exploring the genetic basis of complex diseases at the single cell level." and similar statements in the manuscript misleading.*

**Response:** We appreciated this reviewer with critical point. Our main purpose is not to develop novel methods for expression features of the individual cells, rather, we aim to find joint GWAS and expression association signals at specific cell types from single cell expression data. We are sorry for this confusion to this reviewer, but our work is different from bulk RNA-seq in that we study cell type-specific, joint signals at the network level. Specifically, we used average cell type expression to enable integration of the two types of data (GWAS and scRNA-seq) at the node (i.e., gene) level. The module score is based on node weights. This way of calculating gene-based score is similar to the method implemented by RolyPoly as suggested by the reviewer (point #8):

As for gene co-expression patterns, we actually have implemented it in our previously developed method EW_dmGWAS (Wang et al., 2015), which utilizes both edge and node weights for module search and the edge weight is based on gene co-expression. However, when we tried to transfer the algorithm to scRNA-seq data, there are often too much noise and sparsity. Thus, such work is still undergoing and we will try to release an advanced version of scGWAS in the near future. In addition, our manuscript is currently prepared for not just an introduction of a new method, but the analysis of a large number of GWAS traits in tissue panels using scRNA-seq. If we added co-expression as another feature, our manuscript would have to rewrite substantially, and will add many more figures/tables. To respond to your concern, we have claimed this as one of the limitations of our method (Discussion section, page 37):

“*Second, we ignored the co-expression relationships among gene-gene or protein-protein pairs when searching for modules. Incorporation of more specified gene-gene regulations, such as transcription factors and their target genes, may empower novel discoveries of regulatory module genes*.”

In addition, we have rewritten the statement in the abstract as below:

“*In summary, scGWAS is a powerful tool for exploring the genetic basis of complex diseases at the cell type level using single cell expression data*.”

**Reference**:

Wang Q, Yu H, Zhao Z, Jia P. (2015) EW_dmGWAS: edge-weighted dense module search for genome-wide association studies and gene expression profiles, Bioinformatics, 31(15): 2591-2594

*3)      It is not reported how cell types were defined based on the different scRNA-seq datasets. Given that different studies may have used very different procedures to define cell types it is important to discuss this and to provide guidelines for the users interested in applying the scGWAS tool on a new dataset without annotations.*

**Response**: We thank the reviewer for sharing this point. We did not systematically study how to cluster single cells to cell types because it is out of the scope of this work. The definition of cell types and annotating new cell types have been a hot research topic during the past few years, as extensively shown in the literature. Our manuscript is already very long with many figures and other data, so it is hard for us to develop new method or protocol for cell type annotations from scRNA-seq data. The main purposes of scGWAS are to identify the cell types in which GWAS signals are enriched and to identify the modules that mediate the enrichment, with the assumption that the cell types used as input to scGWAS have already been defined in previous analyses.

In the revised manuscript, the Discussion section, page 37, we added, “*Third, scGWAS does not conduct cell type classification. Users can define cell types using standard strategies and provide scRNA-seq data with annotations to scGWAS.*”

*4)      I found the attempt to map GWAS gene scores and scRNA-seq scores to normal distribution very artificial and ad hoc. For example, as also observed by the authors scRNA-seq data after their proposed procedures is still right skewed. Therefore, additional corrections are necessary downstream, and the entire procedure is composed of a series of ad-hoc and not well justified transformations.*

**Response**: We thank the reviewer for this valuable suggestion. During revision, we have implemented the Box-Cox transformation for scRNA-seq too, and thus, both types of node weights are approximate to the normal distribution. Because of this, we have re-run all the analyses, including 40 traits in 18 scRNA-seq panels. This reanalysis has taken several weeks for us to complete. In the revised manuscripts, all related figures have been updated. The majority of the trait and cell type associations has remained the same, proving the stability and reproducibility of our approach.

*5)      Several thresholds are necessary to define the modules (e.g. r1 and r2) however it is not discussed in detail how these thresholds should be optimally adjusted for a new dataset, but only empirical values are provided in the supplementary material.*

**Response**: We thank the reviewer for this technical point. The two values were selected partially based on experience but mainly based on the actual data. As explained in the manuscript, the parameter r1 determines when the module stops expansion and the parameter r2 determines when the module stops shrinking. We can use the random modules to estimate r1 and r2 because the random modules were generated using the same MEBE algorithm but using randomized node scores. Thus, the random modules represent the null distribution of module scores when the GWAS data and cell type expression data are not related.

For each trait and cell type pairs, we collected the random modules and calculated the increasing ratio of module scores by module size being 3 to 9 (note that very few modules could have a size of 10 or larger). That is, ratio = (m_k_ – m_k-1_)/m_k-1_, where m_k_ is the average module score for modules with size k and k = 4,…,9. As shown in Figure 3 below, when module size increased from 4 to 5, the increase ratio was 12.7% (the vertical line in red); when module size increased from 5 to 6, the ratio was 9.4%; when module size increased from 6 to 7, the ratio was 6.9%; when module size increased from 7 to 8, the ratio was 5.6%; and when module size increased from 8 to 9, the ratio was 3.5%. Thus, r1 = 0.1 would result in modules mostly at size 7 and few modules can keep expanding to a size of 8 or larger. This is consistent with our general understanding about GWAS data and scRNA-seq data because it is unlikely to have a large module where the component genes have both significant GWAS p-values and high cell type expression. With the value of r1 being 0.1, we set r2 to be half the value of r1. Thus, with our settings of r1 and r2, every time when the module includes one more node, the module score increases by at least 10%; and every time the module excludes one node, the module score decreases by at most 5%.

**Figure 3 (Figure S2 in the revised manuscript).** Distribution of module score ratio with different module sizes. The red line indicates the mean ratio for each specific module size.

Although r1 and r2 can be changed, we do not suggest users to change them or “optimally adjust” the values for new datasets. As we have demonstrated in 40 GWAS datasets and 18 scRNA-seq panels, these values were always set as r1 = 0.1 and r2 = 0.05, and the results were reasonable. On our GitHub website, we have added the suggestion for r1 and r2. In the revised manuscript, we have included the analyses above in the supplementary file.

*6)      Virtual module search: this procedure is based on permutations of existing weights. The author report to have at least 100 for each module size, however they then assume a normal distribution  z-scoring the resulting module scores. However, this assumption was not tested and with only 100 permutations it is not clear how they can set the threshold to pm<0.05. Also there are some typos "Virtue" instead of "Virtual" in the main text.*

**Response**: This is a very good suggestion to have more permutations. During revision, we have re-run all analyses and increased the times of virtual search to ensure at least 1000 random modules for each module size. We randomly selected three traits in different cell types and showed the distribution of raw module scores before the z-score normalization. As shown below, for each size with respect to the trait and cell type, we generated at least 1000 random modules and the module scores formed a distribution that was approximate to the normal distribution. It also showed that with the increase of the module size, the raw module scores increased as well, proving the necessity to normalize the raw module scores by size.

**Figure 3.** Demonstration of module score distributions. We used the raw module score for RA, BMI, and asthma for demonstration. The cell types were also randomly selected. The distribution was formed for each specific module size for each pair of trait and cell type.

We apologize for the typo of “virtue”. In the revised manuscript, we have systematically corrected the typos of “virtue” to “virtual”. We have requested a professional editing service to improve the English writing, and acknowledged in the section Acknowledgements.

*7)      Assessment of combined module: the authors used GSEA and in particular NES scores to measure "overall significance" line 51 page 8. However later they state that this score is only used for "presentation purpose". Also, I don't understand the final sentence "Thus there is no data circulation issue". This sentence is also repeated in the discussion*

**Response:** We are sorry for not being clear. This sentence was added to address the potential concern that some users may argue we use GWAS and scRNA-seq data for both module discovery and module evaluation (i.e., data re-use or circulation). In fact, the modules were discovered by using GWAS and scRNA-seq data. Their significance was estimated by using the random modules and the proportional test. Thus, there is no data re-use or circulation issue.

As for NES, we calculated NES for an assessment of the combined network. Because GWAS and scRNA-seq data have already been used in the module discovery step, they should not be used again to assess the significance. Thus, we used NES only to draw the figures (for presentation purpose).

In the revised manuscript, page 37, we rewrote the sentence: “*Thus, the GWAS data and scRNA-seq data were only used for module discovery but not for module evaluation; otherwise, it would raise an issue of circulation use of data.*”

*8)      Previous relevant work is not acknowledged, and the manuscript lacks a reasonable comparison with existing methods: e.g.*  [*https://www.ncbi.nlm.nih.gov/pmc/articles/PMC5896795/*](https://www.ncbi.nlm.nih.gov/pmc/articles/PMC5896795/) *(see in particular Figure 1 for the proposed workflow for RNA+GWAS summary statistics).*
[*https://www.ncbi.nlm.nih.gov/pmc/articles/PMC5673624/*](https://www.ncbi.nlm.nih.gov/pmc/articles/PMC5673624/)

[*https://www.frontiersin.org/articles/10.3389/fgene.2021.637322/full*](https://www.frontiersin.org/articles/10.3389/fgene.2021.637322/full)

[*https://genomemedicine.biomedcentral.com/articles/10.1186/s13073-022-01021-1*](https://genomemedicine.biomedcentral.com/articles/10.1186/s13073-022-01021-1) *and* [*https://www.nature.com/articles/s41467-019-11181-1*](https://www.nature.com/articles/s41467-019-11181-1) *.

Please compare scGWAS with LD score regression, RolyPoly and FUMA.*

**Response**: We thank the reviewer for pointing out these related papers. Some papers are the analysis of GWAS at the tissue level, which is different from our network module-based work using cell types from scRNA-seq. During revision, we have tried our best to compare the three methods, as we were only given 40 days to submit the revised manuscript. In brief, RolyPoly took too long to run the analysis and unfortunately, we had to give up after several trials (see below). For FUMA and LDSC, scGWAS tended to report similar cell types that were also identified by the two methods in most cases. In addition, when the scRNA-seq dataset has multiple similar cell types (e.g., different neuron types), scGWAS is more sensitive than LDSC and FUMA to detect the disease-relevant cell types that were well-known. Most importantly, compared to all three methods, scGWAS has its unique function to identify modules with component genes whose GWAS signals and cell-type expression are both significantly higher than randomly expected.

Specifically, for RolyPoly, the original paper was published in 2017. The R package is no longer accessible by the R website CRAN. We managed to download the archived version and installed it in the R environment anyway. We tried to apply RolyPoly to the traits and panels we used. However, the running time is way too long. As shown below (a screenshot from our attempt), when running RolyPoly, it took nearly 7 hours for one chromosome for one pair of “trait and scRNA-seq” association analysis. That means it would take ~6 days to test one pair of “trait and scRNA-seq” association (7 * 22 chromosomes/24 hours). This was conducted in a workstation with Intel(R) Core(TM) i7-9700K CPU @ 3.60GHz and 32GB RAM. The analysis used 18GB RAM. Due to the long-running time and there are 40 traits by 18 panels, we decided to give up running RolyPoly.

**Figure 4**. Screenshot of running RolyPoly. The left panel shows the running time of each chromosome. The right panel shows the required RAM by RolyPoly.

For FUMA, it is an online system, which means it is convenient for users from any background (e.g., those without a computational background). However, it is also limited to the scRNA-seq panels as provided. Among the 18 panels we have analyzed, only 5 of them were available in FUMA. We selected two psychiatric disorders (schizophrenia and bipolar disorder) and two PsychENCODE panels for comparison, as these disorders have been well-studied for their associated cell types, i.e., various types of neurons. The two PsychENCODE panels were PsychENCODE_developmental, which is panel DER20 in our work containing 35 cell types (8 excitatory neurons, 8 inhibitory neurons, some fetal cell types, and others) and PsychENCODE_adult, which is panel DER22 in our work containing 25 cell types (9 excitatory neurons, 8 inhibitory neurons, and others).

For LDSC, it is a command line tool. Users need to prepare the scRNA-seq panel and provide the list of regions/genes that are specific to the cell type in investigation. In the original work of LDSC, the top 10% regions/genes were suggested. We followed this procedure and selected the top 10% specifically expressed genes per cell type for the DER20 and DER22 panels for comparison. The results from LDSC (-log10 p-value), FUMA (-log10 p-value), and scGWAS (-log10 p-value transformed from the proportional test) were presented in Table 1 (DER20), Table 2 (DER22), and Figure 5 below.

**Table 1.** Disorder and cell type enrichment results from LDSC, FUMA, and scGWAS using the DER20 (PsychENCODE_developmental) dataset [the value is -log10(p-value)].

| **Schizophrenia** | **LDSC** | **FUMA** | **scGWAS** |  | **Bipolar Disorder** | **LDSC** | **FUMA** | **scGWAS** |
| --- | --- | --- | --- | --- | --- | --- | --- | --- |
| Astrocytes | 6.26 | 0 | 0 |  | Astrocytes | 3.69 | NA | 36.18 |
| Endothelial | 9.91 | 0 | 0 |  | Endothelial | 3.96 | 0.03 | 0 |
| Ex1 | 1 | 3.86 | 60.66 |  | Ex1 | 1.12 | 2.04 | >300 |
| Ex2 | 0.93 | 3.66 | >300 |  | Ex2 | 1.53 | 0.93 | >300 |
| Ex3 | 1.52 | 3.15 | >300 |  | Ex3 | 1.14 | 1.25 | >300 |
| Ex4 | 1.03 | 3.82 | >300 |  | Ex4 | 0.82 | 0.99 | 191.9 |
| Ex5 | 0.88 | 3.48 | 165.84 |  | Ex5 | 0.84 | 1.29 | 298.77 |
| Ex6 | 2.57 | 2.7 | 184.2 |  | Ex6 | 1.63 | 1.42 | 302.93 |
| Ex7 | 1.37 | 4.22 | 303.78 |  | Ex7 | 0.88 | 2.05 | >300 |
| Ex8 | 1.2 | 3.54 | 262.73 |  | Ex8 | 1.75 | 2.76 | >300 |
| Fetal_Astrocytes | 5.43 | 0.03 | 0 |  | Fetal_Astrocytes | 3.85 | 0.03 | 0 |
| Fetal_Endothelial | 4.98 | 0 | 0 |  | Fetal_Endothelial | 3.49 | 0 | 0 |
| Fetal_ExN | 6.82 | 0 | 0 |  | Fetal_ExN | 6.58 | 0.02 | 0 |
| Fetal_IntN | 4.65 | 0 | 0 |  | Fetal_IntN | 3.43 | 0.01 | 0 |
| Fetal_IPC | 6.93 | 0 | 0 |  | Fetal_IPC | 4.91 | 0.02 | 0 |
| Fetal_Microglia | 6.17 | 0.01 | 0 |  | Fetal_Microglia | 4.22 | 0.02 | 0 |
| Fetal_NEP | 9.08 | 0 | 0 |  | Fetal_NEP | 5.61 | 0.1 | 0 |
| Fetal_Oligo | 9.67 | 0 | 0 |  | Fetal_Oligo | 6.96 | 0.01 | 0 |
| Fetal_OPC | 5.85 | 0.02 | 0 |  | Fetal_OPC | 2.35 | 0.09 | 0 |
| Fetal_Pericytes | 7.29 | 0 | 0 |  | Fetal_Pericytes | 5.99 | 0 | 0 |
| Fetal_quiescent | 3.59 | 0.05 | 0 |  | Fetal_quiescent | 2.55 | 0.12 | 0 |
| Fetal_replicating | 7.33 | 0 | 0 |  | Fetal_replicating | 3.71 | 0.31 | 0 |
| Fetal_Trans | 12.4 | 0 | 0 |  | Fetal_Trans | 6.22 | 0.01 | 0 |
| In1 | 1.68 | 2.39 | >300 |  | In1 | 2.72 | 1.06 | >300 |
| In2 | 3.11 | 1.4 | 296.23 |  | In2 | 0.9 | 0.95 | >300 |
| In3 | 2.9 | 2.42 | 286.97 |  | In3 | 2.17 | 1.23 | 171.26 |
| In4 | 1.16 | 3.07 | >300 |  | In4 | 0.31 | 1.54 | 178.41 |
| In5 | 3.18 | 2.36 | >300 |  | In5 | 0.4 | 1.84 | 276.78 |
| In6 | 2.14 | 2.89 | 122.31 |  | In6 | 0.29 | 1.21 | >300 |
| In7 | 2.59 | 2.28 | 91.65 |  | In7 | 0.15 | 0.94 | >300 |
| In8 | 2.2 | 2.87 | 204.69 |  | In8 | 1.01 | 0.78 | 95.72 |
| Microglia | 10.23 | 0 | 0 |  | Microglia | 5.27 | 0.17 | 0 |
| Neurons | 2.34 | 0.02 | 0 |  | Neurons | 0.69 | 0.09 | 0 |
| Oligodendrocytes | 5.71 | 0.03 | 0 |  | Oligodendrocytes | 4.16 | 0.07 | 0 |
| OPC | 6.81 | 0 | 0 |  | OPC | 2.72 | 0.04 | 0 |

**Table 2**. Disorder and cell type enrichment results from LDSC, FUMA, and scGWAS using the DER22 (PsychENCODE adult) dataset [the value is -log10(p-value)].

| **Schizophrenia** | **LDSC** | **FUMA** | **scGWAS** |  | **Bipolar Disorder** | **LDSC** | **FUMA** | **scGWAS** |
| --- | --- | --- | --- | --- | --- | --- | --- | --- |
| Astro | 4.87 | 0.88 | 169.44 |  | Astro | 3.86 | 0.02 | 91.16 |
| Endo | 10.36 | 0 | 148.64 |  | Endo | 5.47 | 0.01 | 0 |
| Ex1 | 0.25 | 0.6 | 196.86 |  | Ex1 | 0.05 | 0.58 | >300 |
| Ex2 | 0.45 | 1.13 | 54.45 |  | Ex2 | 0.88 | 0.08 | >300 |
| Ex3e | 1.08 | 0.37 | 125.95 |  | Ex3e | 0.2 | 0.31 | 13.81 |
| Ex4 | 0.36 | 2.06 | 192.59 |  | Ex4 | 0.21 | 0.4 | 218.37 |
| Ex5b | 0.16 | 0.93 | 66.52 |  | Ex5b | 0.02 | 0.43 | 115.84 |
| Ex6a | 1.3 | 0.15 | 87.58 |  | Ex6a | 0.17 | 0.39 | 12.73 |
| Ex6b | 0.46 | 0.71 | 151.89 |  | Ex6b | 0.15 | 0.98 | 80.78 |
| Ex8 | 0.29 | 1.84 | >300 |  | Ex8 | 0.12 | 2.06 | 153.53 |
| Ex9 | 2.92 | 0.04 | 12.29 |  | Ex9 | 2.25 | 0.07 | 0 |
| In1a | 2.03 | 0.47 | 185.06 |  | In1a | 0.38 | 1.65 | 105.29 |
| In1b | 1.41 | 0.4 | >300 |  | In1b | 0.61 | 1.27 | 68.96 |
| In1c | 1.31 | 0.35 | 250.14 |  | In1c | 0.39 | 0.23 | 30.09 |
| In3 | 1.44 | 0.8 | 175.86 |  | In3 | 0.36 | 0.55 | 137.82 |
| In4a | 1.63 | 0.71 | 125.14 |  | In4a | 0.39 | 0.78 | >300 |
| In4b | 2.63 | 0.5 | 122.15 |  | In4b | 0.27 | 1 | 86.63 |
| In6a | 2.35 | 0.43 | 128.75 |  | In6a | 1.43 | 0.17 | 0 |
| In6b | 1.86 | 0.53 | 92.78 |  | In6b | 0.47 | 1.01 | 37.79 |
| In7 | 1.05 | 0.34 | 186.21 |  | In7 | 0.09 | 0.66 | 60.02 |
| In8 | 2.16 | 0.13 | 81.99 |  | In8 | 0.14 | 0.26 | 13.24 |
| Microglia | 9.73 | 0.11 | 201.57 |  | Microglia | 7.77 | 0.03 | 0 |
| Oligo | 7.32 | 1.89 | 124.38 |  | Oligo | 8.62 | 0.14 | 0 |
| OPC | 4.51 | 0.9 | >300 |  | OPC | 3.64 | 0.18 | 0 |
| Per | 4.26 | 1.53 | >300 |  | Per | 3.85 | 0.88 | 46.68 |

**Figure 5.** Venn diagram showing the overlapping cell types among LDSC, FUMA, and scGWAS.

Overall, for the DER20 panel, scGWAS and FUMA showed quite consistent results for schizophrenia but less consistent results for bipolar disorder. For the DER22 panel, scGWAS identified more cell types than LDSC and FUMA. When we examined the extra cells for both disorders (6 for schizophrenia and 15 for bipolar disorder), we found that they were almost exclusively neuron cell types. The 6 unique cell types by scGWAS for schizophrenia in DER22 were Ex1, Ex2, Ex3e, Ex5b, Ex6b, In7. The 15 unique cell types by scGWAS for bipolar disorder in DER22 were: Ex1, Ex2, Ex3e, Ex4, Ex5b, Ex6a, Ex6b, In1b, In1c, In3, In4a, In4b, In6b, In7, and In8. We reasoned that these unique cell types identified by scGWAS should not be false positives but should be authentic signals that were missed by LDSC and FUMA based on the following reasons.

First, it is well-known that schizophrenia and bipolar disorder are associated with different excitatory neurons and inhibitory neurons and such associations have been reported in previous studies. In fact, the results using the DER20 also proved the association. Thus, in DER22, it deserves further investigation when LDSC and FUMA only reported some of the neuron cell types and missed the majority of others.

Second, we examined the strategies used by LDSC and FUMA and we reasoned that they missed some of the neuronal cell types because these two methods use the specifically expressed genes as input and are sensitive to the definition of the “specifically expressed genes”. When defining “cell type specific genes”, the strategy often makes contrast between one cell type of interest versus other cell types. Such a strategy works well in distinguishing cell clusters. However, when applied to define cell type specific genes among the different sub-types of excitatory neurons and inhibitory neurons, this strategy might cancel out some of the important genes shared among neuron cells, because it is well known that these different sub-types of neuron cells are functionally similar to each other than that to non-neuronal cells. We thought that this is the reason that LDSC and FUMA only detected some of the neuronal cell types.

In fact, we and others have noticed this issue in previous tissue-specific enrichment analyses (Finucane et al., 2018; Pei et al., 2019), where tissues of the same group may cancel out the tissue-specific signals when compared together. Finucane et al. defined a concept of “tissue group” and proposed to compare one tissue with those tissues that do not belong to the same tissue group, rather than all other tissues (Finucane et al., 2018). In our previous work CSEA-DB (Dai et al., 2021), we defined cell-type specific genes by considering the sub-type issue and the results showed that nearly all excitatory neurons and inhibitory neurons were associated with schizophrenia and bipolar disorder (Table 3 below), consistent with the results from scGWAS. These results proved that LDSC and FUMA are indeed sensitive to the definition of “cell type specific genes”. In contrast, scGWAS uses average gene expression per cell type and searches for the concordance between GWAS signals and cell type expression levels. The significance is estimated by random modules, rather than by comparison with other cell types. In this regard, scGWAS is more sensitive than LDSC and FUMA to detect cell types that are very similar to each other, such as neuronal cells.

**Table 3**. Cell type enrichment results from CSEA-DB.

|  | Schizophrenia | | | |  | Bipolar Disorder | | | |
| --- | --- | --- | --- | --- | --- | --- | --- | --- | --- |
| Threshold to define significant genes from p(Magma)* | 0.01 | 0.001 | 1.00E-04 | 1.00E-05 |  | 0.05 | 0.01 | 0.001 | 1.00E-04 |
| Excitatory neuron 1 | 1.09E-13 | 3.32E-11 | 9.55E-11 | 4.27E-07 |  | 2.09E-16 | 1.68E-07 | 0.000103 | 0.00094 |
| Excitatory neuron 3a | 9.19E-16 | 3.48E-12 | 1.44E-07 | 9.99E-05 |  | 1.47E-10 | 0.000116 | 0.00254 | 0.00212 |
| Excitatory neuron 3b | 8.47E-09 | 1.98E-07 | 4.87E-06 | 0.00238 |  | 3.48E-13 | 3.08E-07 | 0.00141 | 0.00094 |
| Excitatory neuron 3c | 0.00212 | 0.00222 |  |  |  | 2.53E-05 | 0.00234 | 0.0202 | 0.0183 |
| Excitatory neuron 3d | 2.39E-08 | 5.89E-06 | 0.000565 |  |  | 7.93E-05 | 0.000291 |  |  |
| Excitatory neuron 4 | 1.85E-15 | 1.61E-12 | 1.44E-08 | 1.11E-05 |  | 1.36E-09 | 0.000116 | 0.0483 |  |
| Excitatory neuron 5a | 2.81E-07 | 1.99E-06 | 0.000191 | 0.000383 |  | 2.57E-11 | 9.02E-08 | 0.0126 | 0.0183 |
| Excitatory neuron 5b | 4.99E-09 | 3.44E-06 | 1.77E-05 | 9.99E-05 |  | 8.69E-07 | 0.000291 | 0.0317 |  |
| Excitatory neuron 6a | 0.0182 |  | 0.0207 |  |  | 0.0182 |  |  | 0.00935 |
| Excitatory neuron 6b | 1.43E-08 | 4.52E-05 | 0.000565 |  |  | 0.000459 | 7.16E-05 | 0.00254 | 0.0183 |
| Excitatory neuron 8 | 3.33E-05 | 0.00463 | 0.0207 |  |  | 0.000164 | 0.00987 |  |  |
| Inhibitory neuron 1a |  | 0.0408 |  |  |  | 0.000879 |  | 0.0317 |  |
| Inhibitory neuron 1b | 0.00043 | 0.00323 | 0.0141 |  |  | 0.00163 |  | 0.0202 |  |
| Inhibitory neuron 1c | 0.00504 | 0.0173 | 0.00155 |  |  | 0.00871 |  | 0.0202 |  |
| Inhibitory neuron 2 | 0.0441 |  | 0.0422 |  |  | 0.0143 |  | 0.0126 |  |
| Inhibitory neuron 3 | 0.00862 |  | 0.0298 |  |  |  | 0.0339 | 0.00444 | 0.00935 |
| Inhibitory neuron 4a | 0.00381 | 0.0311 |  |  |  | 0.0022 | 0.00158 | 0.00444 | 0.0339 |
| Inhibitory neuron 4b | 9.90E-06 | 0.000288 | 0.00155 | 0.0294 |  | 3.25E-06 | 5.44E-06 | 0.0126 |  |
| Inhibitory neuron 6a | 0.00212 |  |  |  |  | 0.0285 | 0.0254 |  |  |
| Inhibitory neuron 6b | 7.61E-13 | 3.58E-07 | 0.000191 | 0.0188 |  | 7.93E-05 | 0.00105 | 0.000207 | 0.00212 |
| Inhibitory neuron 7 | 1.49E-05 | 0.00222 |  |  |  |  |  |  |  |
| Inhibitory neuron 8 | 2.24E-05 | 0.000288 | 0.0298 |  |  | 0.00871 | 0.0339 | 0.0317 | 0.0339 |
| Endothelial cell |  |  |  |  |  |  |  |  |  |
| Pericytes |  |  |  |  |  |  |  |  |  |
| Astrocyte | 0.0288 |  |  |  |  |  |  |  |  |
| Oligodendrocyte | 0.0441 | 0.00463 | 0.00942 | 0.00415 |  |  |  |  |  |
| Oligodendrocyte precursor cell | 0.0182 | 0.00656 |  |  |  |  |  |  |  |
| Microglia |  |  |  | 0.0117 |  |  |  |  |  |

*For each trait, CSEA-DB uses different threshold for p(Magma) to define trait-associated genes. For schizophrenia, four sets of trait-associated genes were assessed for association with cell types from the DER22 panel: genes with p(Magma) < 0.01, p(Magma) < 0.001, p(Magma) < 1E-4, and p(Magma) < 1E-5. For bipolar disorder, the four sets of trait-associated genes were defined as genes with p(Magma) < 0.05, p(Magma) < 0.01, p(Magma) < 0.001, and p(Magma) < 1E-4 (no genes at p<1E-5). The value in each cell is the p-value from the cell-type enrichment test implemented in CSEA-DB (Dai et al., 2021).

Thus, we considered our strategy of using average expression and estimating the concordance between GWAS and cell-type expression is more sensitive than the methods that are built on the definition of cell-type-specific genes. In the revised manuscript, we have claimed the works of RolyPoly, FUMA, LDSC, as well as our own work CSEA-DB and deCS. In addition, we included the comparison results in the supplementary file.

References:

Finucane, H.K. *et al.* Heritability enrichment of specifically expressed genes identifies disease-relevant tissues and cell types. *Nat Genet* **50**, 621-629 (2018).

Pei, G., Dai, Y., Zhao, Z. & Jia, P. deTS: tissue-specific enrichment analysis to decode tissue specificity. Bioinformatics 35, 3842-3845 (2019).

Dai, Y. et al. CSEA-DB: an omnibus for human complex trait and cell type associations. Nucleic Acids Res 49, D862-D870 (2021).

Pei, G. et al. deCS: A tool for systematic cell type annotations of single-cell RNA sequencing data among human tissues. Genomics Proteomics Bioinformatics (2022).

*9)      The software package is poorly documented and only a succinct example is provided on GitHub. The documentation needs to be expanded and the expected outputs described. Also, to ensure reproducibility it is important to show how the package was used to obtain the results presented in the manuscript and share the additional R scripts used.*

**Response**: We thank the reviewer for this suggestion. We have substantially enhanced the documentation in GitHub by providing the package, preprocessing codes, post-analysis codes, as well as a work-through example. We also provided the supporting data and related codes for reproducing the results and figures in the manuscript.

**Second round of review**

**Reviewer 1**

The authors have carefully addressed my previous comments. I am satisfied with the changes made and have no further suggestions.

**Reviewer 2**

I think the manuscript was substantially improved and I praise the authors for their efforts in addressing my concerns. However, before publications it is necessary to fully address some of the points raised during the first revision as suggested below.

1) SNP to gene mapping is limited to proximal region ( main concern #1 from my past report)

I think adding a short sentence in the method section to address this is not sufficient. I propose two minor changes:

- Please add a note in the discussion section highlighting the limitation of the current approach.

- Regarding "we also leave the option open for users to provide their own way of mapping SNPs to genes and calculating gene-based scores based on any regulatory regions, including enhancers." In the method section and documentation I was not able to find how this can be accomplished. Please provide sufficient details so this is clear from the manuscript and documentation.

2) I think the new statement added proposed by the author doesn't fully address my concern. Given that the proposed method name (scGWAS) may mislead potential users the manuscript should state explicitly that the all the estimates are not at single cell level however based on aggregation procedure (pseudo-bulk expression levels) as proposed by previously developed methods as RolyPoly.

I suggest to add this statement in the discussion section: "Despite our method can take in input single-cell level expression data, scGWAS is not able to provide information on individual cells. Rather we leverage aggregation procedures (e.g. pseudo-bulk expression levels based on clusters or external annotations) as proposed by previously developed methods (cite RolyPoly)"

3) Regarding the statement: "Third, scGWAS does not conduct cell type classification. Users can define cell types using standard strategies and provide scRNAseq data with annotations to scGWAS."

I suggest to modify it as follow:

"Third, scGWAS is not and end-to-end pipeline, for example it doesn't include any module to preprocess, normalize and batch correct single-cell data nor to conduct cell type classification/clustering. Therefore users need to use available single-cell pipelines (cite seurat, scanpy) to define cell types and to provide annotations to scGWAS."

4) In the new sentence response to point 7) I suggest to revise "issue of circulation of data" with "overestimation of performance based on reusing the same data for two different tasks"

5) I praise the author for the systematic comparison of scGWAS with RolyPoly, FUMA and LDSC. However I was surprised to see that this analysis was not described in the main result section. Please briefly present this comparative analyses in the result section of the main text. I suggest to add a new subsection called "Comparison of scGWAS with similar methods" and to report the details in a dedicated supplementary note containing the new figures, tables and an adaptation of the text presented in the response to the reviewers document.

**Authors Response**

**Point-by-point responses to the reviewers’ comments:**

Reviewer #1: The authors have carefully addressed my previous comments. I am

satisfied with the changes made and have no further suggestions.

*Response: We thank Reviewer #1’s positive comments on our revised manuscript.*

Reviewer #2: I think the manuscript was substantially improved and I praise the authors for their efforts in addressing my concerns. However, before publications it is necessary to fully address some of the points raised during the first revision as suggested below.

1) SNP to gene mapping is limited to proximal region (main concern #1 from my past

report) I think adding a short sentence in the method section to address this is not sufficient. I

propose two minor changes:

- Please add a note in the discussion section highlighting the limitation of the current

approach.

*Response: We added a note in the Discussion section to highlight the limitation (page*

*14, the last paragraph):*

*“This strategy is limited to proximal regions and might miss important SNP-gene relationships.”*

- Regarding "we also leave the option open for users to provide their own way of mapping SNPs to genes and calculating gene-based scores based on any regulatory regions, including enhancers." In the method section and documentation, I was not able to find how this can be accomplished. Please provide sufficient details so this is clear from the manuscript and documentation.

*Response: We are sorry for not being clear on this statement. We now added a sentence in the main manuscript (page 15, the first paragraph):*

*“In practice, users can map SNPs to genes using customized strategies and then provide the resultant gene-based scores to scGWAS for the follow up analyses.”*

*In the documentation*

*(https://github.com/bsml320/scGWAS/blob/main/analysis/explanation_configure.md ), we added a note for gene-based scores as provided in the file “gwas_node_file”.*

2) I think the new statement added proposed by the author doesn't fully address my concern. Given that the proposed method name (scGWAS) may mislead potential users the manuscript should state explicitly that the all the estimates are not at single cell level however based on aggregation procedure (pseudo-bulk expression levels) as proposed by previously developed methods as RolyPoly. I suggest to add this statement in the discussion section: "Despite our method can take in input single-cell level expression data, scGWAS is not able to provide information on individual cells. Rather we leverage aggregation procedures (e.g. pseudo-bulk expression levels based on clusters or external annotations) as proposed by previously developed methods (cite RolyPoly)"

*Response: In response to your concern, we now added this statement in the main manuscript (page 15, the last paragraph):*

*“Fourth, despite that our method takes single-cell level expression data as the input, scGWAS is not able to provide information on individual cells. Rather we leverage aggregation procedures (e.g., pseudo-bulk expression levels based on clusters or external annotations) as proposed by previously developed methods11.”*

3) Regarding the statement: "Third, scGWAS does not conduct cell type classification.

Users can define cell types using standard strategies and provide scRNAseq data with annotations to scGWAS."

I suggest to modify it as follow: "Third, scGWAS is not and end-to-end pipeline, for example it doesn't include any module to preprocess, normalize and batch correct single-cell data nor to conduct cell type classification/clustering. Therefore users need to use available single-cell pipelines (cite seurat, scanpy) to define cell types and to provide annotations to scGWAS."

*Response: We added this statement in the main manuscript (page 15, the last paragraph):*

*“Third, scGWAS is not an end-to-end pipeline. For example, it does not include any module to preprocess, normalize, and batch correct single-cell RNA-seq data, nor to conduct cell type classification/clustering. Therefore, users need to use available single-cell pipelines, such as Seurat35 and Scanpy36, to define cell types and then to provide the annotations to scGWAS.”*

4) In the new sentence response to point 7) I suggest to revise "issue of circulation of data" with "overestimation of performance based on reusing the same data for two different tasks"

*Response: We have revised the sentence according to your suggestion (page 15, the last paragraph): “it would raise an issue of overestimation of performance based on reusing the same data for two different tasks.”*

5) I praise the author for the systematic comparison of scGWAS with RolyPoly, FUMA and LDSC. However I was surprised to see that this analysis was not described in the main result section. Please briefly present this comparative analyses in the result section of the main text. I suggest to add a new subsection called "Comparison of scGWAS with similar methods" and to report the details in a dedicated supplementary note containing the new figures, tables and an adaptation of the text presented in the response to the reviewers document.

*Response: We have added a new subsection in the main manuscript to describe the comparison of scGWAS with other tools (page 6, the last paragraph): “Comparison of scGWAS with similar methods”.*
